# Clinical AI-assisted FXR prioritization and WADDAICA-guided design of a berberine-inspired analogue for MASH: integrated computational and experimental validation

**DOI:** 10.3389/fphar.2026.1862990

**Published:** 2026-07-23

**Authors:** Hui Yao, Yifeng Zhou, Huijie Zhang, Man Ni, Yang Liu, Yuan Zhou

**Affiliations:** 1 Department of Geriatric Medicine, Wuhu City Second People’s Hospital (Affiliated Wuhu Hospital of East China Normal University), Wuhu, Anhui, China; 2 Department of Neurology, The Affiliated Chuzhou Hospital of Anhui Medical University (The First People’s Hospital of Chuzhou), Chuzhou, Anhui, China; 3 Xuzhou Medical University, XuZhou, Jiangsu, China; 4 Outpatient Clinic, The Affiliated Chuzhou Hospital of Anhui Medical University (The First People’s Hospital of Chuzhou), Chuzhou, Anhui, China

**Keywords:** artificial intelligence, berberine analogue, drug discovery, FXR, MASH

## Abstract

**Background:**

Metabolic dysfunction-associated steatotic liver disease (MASLD) and metabolic dysfunction-associated steatohepatitis (MASH), lack effective targeted therapies despite increasing global prevalence. The farnesoid X receptor (FXR) is a key regulator of hepatic lipid metabolism, inflammation, and fibrosis, making it a promising therapeutic target. However, existing FXR modulators are associated with limited efficacy and adverse effects.

**Objective:**

This study aimed to establish a sequential clinical-to-molecular framework combining clinical AI-assisted liver-disease feature learning, FXR target prioritization, WADDAICA-guided molecular design, and experimental validation of a berberine-inspired analogue.

**Methods:**

An SE attention-based Transformer model was trained on the Indian Liver Patient Dataset only for liver-disease classification and clinical feature-prioritization analysis. The model output consisted of disease-classification probability and feature-importance information; it was not used for compound optimization. Molecular design of the berberine-inspired analogue was performed independently using the WADDAICA platform.

**Results:**

The AI model demonstrated excellent performance (AUC = 0.9998), identifying clinically relevant metabolic markers linked to FXR pathways. The WADDAICA-designed berberine-inspired analogue showed favorable predicted interactions with FXR, supported by MD and DFT analyses. Favorable pharmacokinetic properties, including high gastrointestinal absorption and low blood–brain barrier permeability, were observed. *In vitro*, the compound significantly improved cell viability, reduced lipid accumulation, and modulated key molecular markers, including upregulation of FXR and PPARα and downregulation of SREBP-1c, TNF-α, IL-6, and fibrotic markers.

**Conclusion:**

This study presents a multi-scale, AI-integrated pipeline linking clinical prediction with molecular design and biological validation. The WADDAICA-designed berberine-inspired analogue showed FXR-associated molecular changes and preliminary cytoprotective and anti-steatotic effects in an FFA-induced HepG2 model. These findings support further investigation of its putative FXR-mediated mechanism of action using dedicated receptor functional assays.

## 1 Introduction

Metabolic dysfunction-associated steatotic liver disease (MASLD), formerly referred to as non-alcoholic fatty liver disease (NAFLD), represents one of the most prevalent chronic liver disorders globally, affecting approximately 25%–30% of the adult population and closely associated with obesity, insulin resistance, and metabolic syndrome (Younossi et al., 2016; Eslam et al., 2020; Powell et al., 2021). Its progressive form, metabolic dysfunction-associated steatohepatitis (MASH), previously referred to as non-alcoholic steatohepatitis (NASH), is characterized by hepatic steatosis, inflammation, hepatocellular injury, and fibrosis, which may ultimately progress to cirrhosis and hepatocellular carcinoma (Friedman et al., 2018; Estes et al., 2018). Despite the increasing burden of MASLD/MASH, effective pharmacological interventions remain limited, and currently available therapies show variable efficacy and safety profiles, emphasizing the need for novel therapeutic strategies (Younossi et al., 2021; Perazzo and Dufour, 2017).

The farnesoid X receptor (FXR; NR1H4), a bile acid-activated nuclear receptor, has emerged as a central regulator of hepatic metabolic homeostasis. FXR-associated pathway modulation suppresses *de novo* lipogenesis through inhibition of sterol regulatory element-binding protein 1c (SREBP-1c), enhances fatty acid oxidation via peroxisome proliferator-activated receptor alpha (PPARα), and regulates bile acid synthesis through cholesterol 7α-hydroxylase (CYP7A1) (Lefebvre et al., 2009; Zhang and Edwards, 2008). In addition, FXR also exerts anti-inflammatory effects and may influence fibrosis-associated pathways by modulating cytokine production and extracellular matrix remodeling (Chiang, 2017; Han, 2018). Consequently, FXR has been widely investigated as a therapeutic target for MASH; however, clinically available FXR modulators, such as obeticholic acid, are associated with adverse effects, including pruritus and dyslipidemia, and exhibit inconsistent clinical outcomes (Younossi et al., 2021; Younossi et al., 2019). These limitations highlight the need for improved compounds predicted to interact with FXR-associated pathways with enhanced efficacy and safety.

Natural products have historically served as a rich source of bioactive molecules for metabolic and inflammatory diseases. Among these, berberine, an isoquinoline alkaloid isolated from *Coptis* and *Berberis* species, has demonstrated a broad spectrum of pharmacological activities, including lipid-lowering, anti-inflammatory, and insulin-sensitizing effects (Tillhon et al., 2012; Och et al., 2022). Berberine has been reported to ameliorate hepatic steatosis and metabolic dysfunction through modulation of multiple signaling pathways, including AMP-activated protein kinase (AMPK), lipid metabolism, and inflammatory cascades (Imenshahidi and Hosseinzadeh, 2019; Sun et al., 2017). Emerging evidence further suggests that berberine may influence FXR-related pathways, contributing to its hepatoprotective effects (Sun et al., 2017; Shu et al., 2021). However, its clinical application is limited by poor oral bioavailability, rapid metabolism, and lack of target specificity, necessitating rational structural optimization to enhance its therapeutic potential (Tillhon et al., 2012; Wang et al., 2017).

Recent advances in artificial intelligence (AI) have significantly accelerated drug discovery by enabling the integration of complex clinical, molecular, and chemical datasets. Deep learning models, particularly Transformer-based architectures, have demonstrated superior performance in capturing nonlinear relationships and long-range dependencies in biomedical data (Jumper et al., 2021; Madan et al., 2024). Attention mechanisms, including squeeze-and-excitation (SE) modules, further enhance model interpretability by dynamically prioritizing relevant features, thereby facilitating the identification of disease-associated biomarkers and pathways (Madan et al., 2024; Hu et al., 2018). Despite these advances, most studies have focused either on predictive modeling or molecular design independently, with limited efforts to integrate AI-driven clinical insights with downstream target identification and therapeutic optimization in a unified framework (Jumper et al., 2021; Chen et al., 2018).

To address these limitations, the present study proposes an integrated, multi-scale framework that combines AI-based disease prediction, computational drug design, and experimental validation. An SE attention-based Transformer model was first employed to identify key clinical features associated with liver disease progression. These AI-derived insights were subsequently translated into biological target prioritization, leading to the identification of FXR as a central regulatory node. A library of natural compounds was screened for FXR binding, and berberine was selected as a lead candidate for structural optimization.

The WADDAICA-designed berberine-inspired analogue was evaluated using molecular docking, molecular dynamics (MD) simulations, density functional theory (DFT) analysis, and *in silico* pharmacokinetic profiling to assess binding affinity, structural stability, electronic properties, and drug-likeness. Experimental validation was performed in an *in vitro* MASH-like HepG2 model to assess cytoprotective, anti-steatotic, anti-inflammatory, and modulation of fibrosis-associated markers. Finally, an integrated mechanistic model was developed to elucidate FXR-mediated therapeutic action.

The novelty of this study lies in the development of a sequential translational workflow rather than a single end-to-end compound-optimization model. In the first step, an SE attention-based Transformer model was trained on a clinical liver-disease dataset to classify liver-disease status and identify influential biochemical variables associated with liver dysfunction. This model was used only for diagnostic feature learning and biological interpretation. It was not used to generate, rank, or optimize molecular structures. In the second step, FXR was prioritized as a mechanistically relevant target by integrating the clinical feature-learning results with established biological evidence linking FXR to bile-acid metabolism, lipid regulation, inflammation, and fibrosis. Compound design was then performed independently using the WADDAICA molecular design platform, followed by docking, molecular dynamics simulation, ADMET prediction, chemical characterization, and *in vitro* validation. Thus, the clinical AI model supported target-context interpretation, whereas WADDAICA was responsible for molecular design and optimization.

## 2 Materials and methods

### 2.1 Study design

A sequential clinical-to-molecular workflow was developed in this study. The workflow consisted of two technically distinct components: clinical AI-based feature learning and independent structure-based molecular design.

First, an SE attention-based Transformer model was trained using the Indian Liver Patient Dataset. This dataset contains demographic and biochemical liver-function variables and is suitable for liver-disease classification and clinical feature-prioritization analysis. Therefore, the purpose of training the Transformer model was to classify liver-disease status and identify influential clinical variables associated with liver dysfunction. The input variables included age, sex, total bilirubin, direct bilirubin, alkaline phosphatase, alanine aminotransferase, aspartate aminotransferase, total proteins, albumin, and albumin/globulin ratio. The model output was a binary liver-disease prediction together with feature-importance information derived from the attention and SE recalibration mechanisms.

Importantly, the Transformer model was not used for compound generation or molecular optimization. The Indian Liver Patient Dataset does not contain molecular structures, ligand-target activity labels, receptor-binding affinities, pharmacokinetic endpoints, or chemical descriptors. Therefore, it is not suitable for training a compound-optimization model. In the present study, its role was restricted to diagnostic classification, clinical feature learning, and biological interpretation of liver-dysfunction-related variables.

Second, FXR was prioritized as a disease-relevant molecular target based on the combination of clinical feature interpretation and established biological evidence. The Transformer-derived feature-importance results highlighted liver-dysfunction markers such as bilirubin fractions, albumin, and liver enzymes, which are biologically connected to hepatic metabolism and bile-acid regulation. FXR was therefore selected because of its well-established role in bile-acid homeostasis, lipid metabolism, inflammatory regulation, and fibrosis-associated signaling.

Third, molecular design was performed independently using the WADDAICA platform. Berberine was selected as a natural-product lead based on its reported hepatoprotective and metabolic regulatory effects. WADDAICA was used to generate and prioritize berberine-inspired analogues using chemical-structure, physicochemical, receptor-interaction, and drug-likeness criteria. The selected analogue was subsequently evaluated using molecular docking, molecular dynamics simulation, MM-GBSA analysis, DFT calculations, ADMET prediction, chemical characterization, and experimental validation in an FFA-induced HepG2 steatosis/MASH-like model.

Thus, the diagnostic Transformer model and the WADDAICA molecular-design platform served different purposes. The Transformer model supported clinical feature learning and target-context interpretation, whereas WADDAICA was used for compound design and optimization.

**FIGURE 1:**
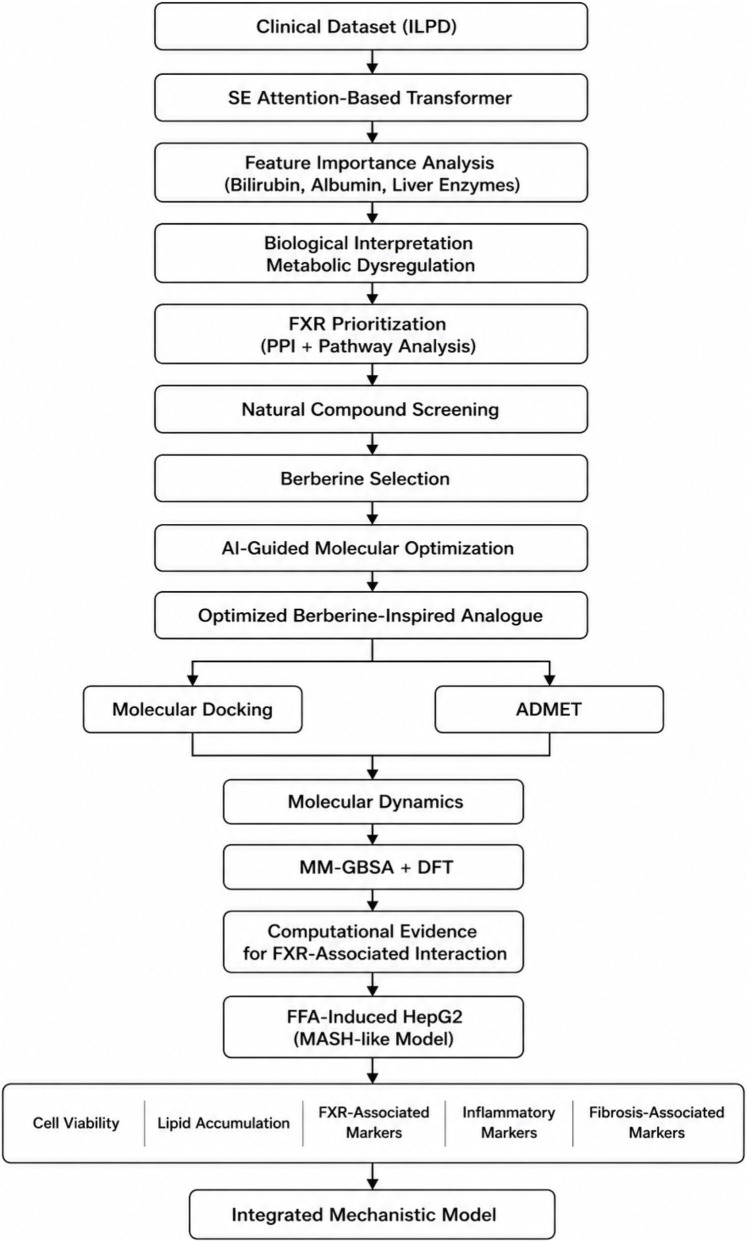
Integrated AI-driven workflow for the identification and evaluation of an FXR-associated therapeutic candidate for MASH. Clinical data from the Indian Liver Patient Dataset (ILPD) were analyzed using an SE attention-based Transformer model to identify key disease-associated features, including bilirubin, albumin, and liver enzyme markers. Biological interpretation of these AI-derived features supported prioritization of the farnesoid X receptor (FXR) through protein–protein interaction (PPI) and pathway analyses. Following natural compound screening, berberine was selected as a lead scaffold and subjected to AI-guided molecular optimization to generate an WADDAICA-designed berberine-inspired analogue. The WADDAICA-designed analogue was subsequently evaluated using molecular docking, ADMET prediction, molecular dynamics (MD) simulations, MM-GBSA binding free energy calculations, and density functional theory (DFT) analysis. Computational findings were then assessed in an FFA-induced HepG2 MASH-like model through measurements of cell viability, lipid accumulation, FXR-associated markers, inflammatory markers, and fibrosis-associated markers. The integrated workflow illustrates the connection between clinical prediction, target prioritization, compound optimization, computational evaluation, and experimental validation, culminating in the development of a mechanistic model describing the potential involvement of FXR-associated pathways in MASH.

The overall workflow of the study, integrating AI-based prediction, computational modeling, and experimental validation, is illustrated in Figure 1. The workflow consisted of.

- Clinical data modeling using an SE attention-based Transformer,
- Biological identification of FXR as a key metabolic regulator,
- WADDAICA-guided design of a berberine-inspired analogue, and
- Validation of therapeutic efficacy *in vitro*.

## 3 Clinical dataset and preprocessing

### 3.1 Data source

Clinical data were obtained from the Indian Liver Patient Dataset (ILPD), accessed via Kaggle and originally curated from the UCI Machine Learning Repository. The dataset includes 583 subjects (416 liver disease cases and 167 controls) with features comprising age, gender, total bilirubin, direct bilirubin, alkaline phosphatase, alanine aminotransferase, aspartate aminotransferase, total proteins, albumin, and albumin/globulin ratio. Although not explicitly labeled for MASH, these biomarkers reflect metabolic liver dysfunction and are closely associated with FXR-regulated pathways.

This dataset was used only for liver-disease classification and clinical feature-learning analysis. It was not used for compound optimization because it does not include molecular structures, ligand-target bioactivity values, binding-affinity data, pharmacokinetic endpoints, or chemical descriptors.

### 3.2 Preprocessing

This process addresses missing values using techniques such as mean or median imputation, as well as more advanced strategies when required. Numerical features (e.g., bilirubin and albumin) were normalized using standardization or min–max scaling to ensure all variables were mapped onto a common scale.

Missing numerical values were imputed using median imputation. Categorical variables (e.g., gender) were encoded using one-hot encoding. Numerical variables were normalized before model training using either z-score standardization or min-max normalization, The dataset was split into training (70%), validation (15%), and testing (15%) sets using stratified sampling to preserve class balance. Feature scaling parameters were fitted on the training set and applied to validation and test sets to prevent data leakage.

### 3.3 Statistical feature characterization

Statistical feature extraction transforms raw clinical data into informative representations that enhance predictive model performance. Key statistical measures, including mean, standard deviation, skewness, and correlation, were computed for each clinical variable, including bilirubin and albumin. The mean provides an overall indicator of liver function, while standard deviation and skewness quantify variability and asymmetry in the data distribution. Correlation analysis identifies relationships among biomarkers, offering insight into disease progression patterns. Collectively, these statistical features improve model accuracy and assist clinicians in making timely, informed decisions regarding liver disease management.

These features provided insight into metabolic variability associated with liver dysfunction.

### 3.4 SE attention-based transformer model

**FIGURE 2:**
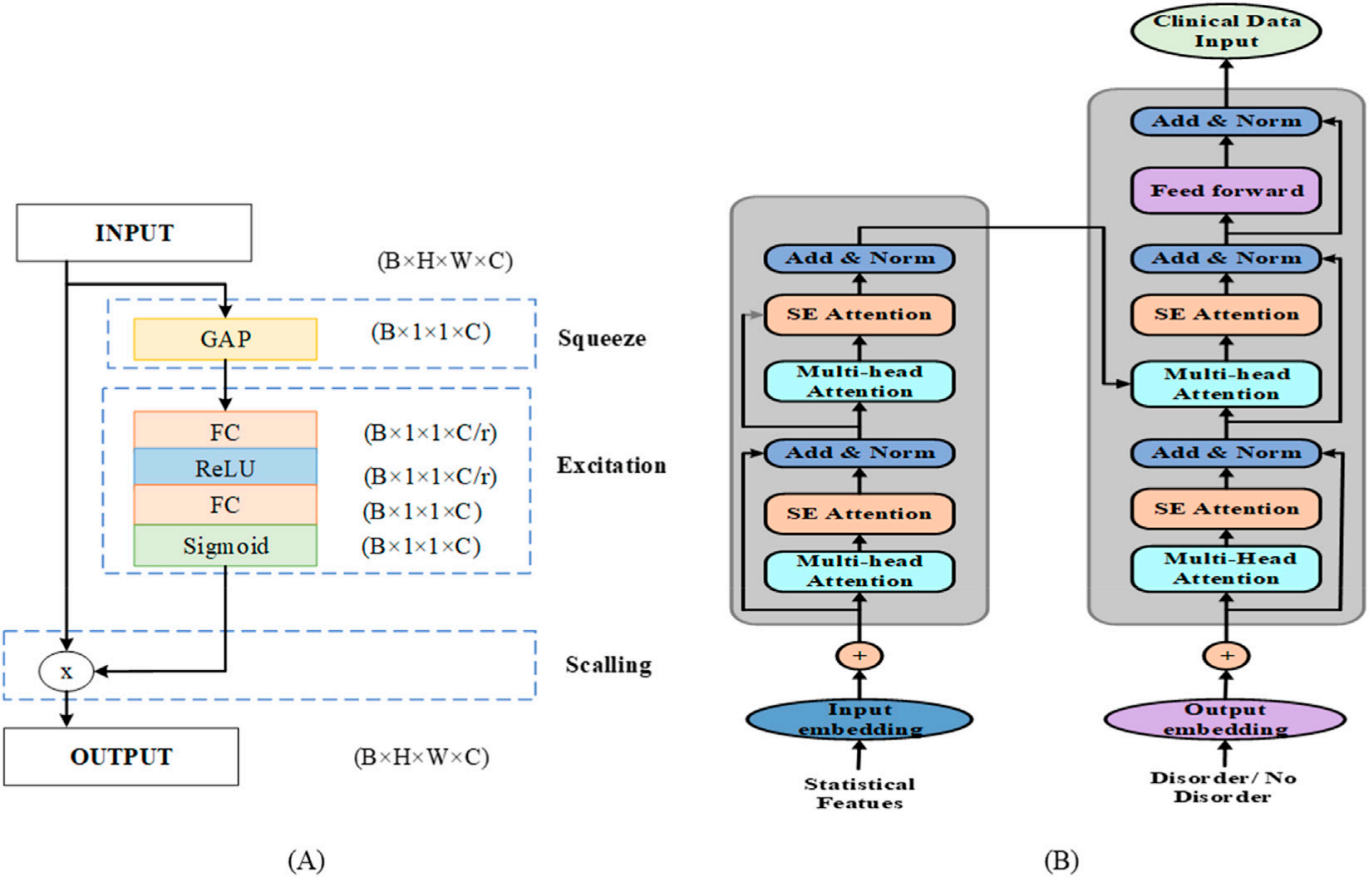
SE attention-based Transformer architecture for liver disease prediction and feature recalibration **(A)** Squeeze-and-Excitation (SE) attention mechanism used for channel-wise feature recalibration. Global average pooling (GAP) is applied to the input feature map (B × H × W × C) to generate channel descriptors, followed by a two-layer fully connected (FC) network with ReLU and sigmoid activations. The resulting weights are used to scale the original feature map, enabling adaptive emphasis of informative clinical features. **(B)** SE attention-based Transformer model for liver disease prediction. Input clinical features are embedded and processed through stacked Transformer encoder blocks incorporating multi-head attention and SE attention modules, each followed by residual connections and normalization (Add & Norm). The architecture captures complex feature interactions and outputs a classification prediction (disease vs no disease).

The architecture of the SE attention-based Transformer model used for liver disease prediction is shown in Figure 2A. The SE Attention-Based Transformer Architecture leverages squeeze-and-excitation (SE) attention mechanisms integrated with a Transformer model to identify and prioritize the most predictive features of liver disease progression. This architecture employs a multitask/identity setup in which the self-attention layers capture complex interrelations among clinical variables, while the SE attention mechanism recalibrates the importance of each feature, enabling the model to assign higher weights to critical markers such as bilirubin and albumin. By combining these components, the model not only enhances prediction accuracy but also provides interpretability by highlighting which clinical features drive disease progression. This hybrid approach facilitates robust, time-aware predictions of liver disease outcomes and supports clinical decision-making. The predictive model was implemented using PyTorch (version 1.12) and trained on a system equipped with an NVIDIA GPU (CUDA-enabled).

Clinical features were transformed into numerical vectors and embedded into a 128-dimensional feature space. The model architecture consisted of two Transformer encoder layers, each with four attention heads and a hidden dimension of 256. Dropout regularization was applied with a rate of 0.3 to reduce overfitting. This mechanism allows the model to focus on the most relevant liver disease markers, such as bilirubin and albumin, while down-weighting less informative features, thereby enhancing prediction accuracy (Figure 2B).

The model was trained using the Adam optimizer with a learning rate of 0.0001. Binary cross-entropy loss was used as the objective function. Training was conducted with a batch size of 32 for up to 100 epochs, with early stopping applied based on validation loss using a patience threshold of 10 epochs.

All experiments were conducted with a fixed random seed (42) to ensure consistent results across runs. The model output was a binary classification representing the presence or absence of liver disease. Performance evaluation was conducted using metrics including accuracy, precision, recall, F1-score, and area under the receiver operating characteristic curve.

The model output was limited to liver-disease classification probability and feature-importance information. It did not output WADDAICA-designed analogues, molecular structures, docking scores, ADMET properties, or synthetic candidates.

## 4 Biological target identification

### 4.1 Protein–protein interaction and pathway analysis

To identify biologically relevant targets associated with liver dysfunction, the farnesoid X receptor (FXR, NR1H4) was selected based on its established role in bile acid regulation, lipid metabolism, and inflammatory signaling.

Protein–protein interaction analysis was performed using the STRING database (version 11.5), with *Homo sapiens* specified as the organism. A confidence score threshold of 0.7 was applied to include only high-confidence interactions supported by experimental evidence or curated databases.

The resulting interaction network was analyzed to identify key signaling partners and pathways associated with FXR-mediated metabolic regulation.

### 4.2 Protein structure preparation

The crystal structure of FXR ligand-binding domain (PDB ID: 6A5W) was retrieved from the Protein Data Bank (https://www.rcsb.org/). Protein preparation was performed using the Schrödinger Protein Preparation Wizard, including hydrogen addition, assignment of protonation states at pH 7.4, removal of non-essential molecules, and energy minimization using the OPLS-2005 force field.

Potential ligand-binding sites were identified using the PrankWeb server, and the highest-ranked binding pocket based on probability and structural accessibility was selected for subsequent docking studies.

### 4.3 Ligand selection and screening

A library of 30 natural compounds with reported hepatoprotective and anti-inflammatory properties was compiled from the PubChem database.

Chenodeoxycholic acid (CDCA) was used as an endogenous FXR ligand, and obeticholic acid (OCA) was included as a clinically validated Putative FXR modulator for benchmarking.

Molecular docking was performed using PyRx (AutoDock Vina backend, version 1.2.0). The docking grid was defined with a center at coordinates (13.767, −14.0696, −14.217) and dimensions of 25 × 25 × 25 Å. The exhaustiveness parameter was set to 8.

Ligand structures were prepared by energy minimization prior to docking. The top-ranked binding poses were selected based on binding affinity scores.

Drug-likeness and physicochemical properties were evaluated using SwissADME, while toxicity predictions were performed using ProTox-II. Compounds with unfavorable pharmacokinetic or toxicity profiles were excluded from further analysis.

## 5 WADDAICA-guided design of a berberine-inspired analogue

Following clinical feature learning and FXR target prioritization, molecular design was performed independently using the WADDAICA molecular design platform. The SE attention-based Transformer model trained on the clinical dataset was not used for compound optimization. Instead, WADDAICA was used as a separate molecular design tool to generate and prioritize berberine-inspired analogues based on chemical-structure modification, predicted receptor compatibility, physicochemical properties, and drug-likeness criteria.

The optimization workflow employed the molecular design and lead-optimization modules to generate structurally related analogues through AI-assisted scaffold modification and functional group substitution. Structural modifications were guided by predicted improvements in receptor interaction, physicochemical properties, and drug-likeness parameters.

Candidate molecules generated by the platform were automatically evaluated using multi-parameter criteria including predicted binding compatibility with the FXR ligand-binding pocket, hydrogen-bonding potential, lipophilicity, molecular weight, topological polar surface area (TPSA), and compliance with drug-likeness rules. Candidate structures showing unfavorable physicochemical properties or poor predicted pharmacokinetic characteristics were excluded from further consideration (Bai et al., 2021).

The final WADDAICA-designed berberine-inspired analogue was selected based on its overall balance of predicted receptor interaction potential, physicochemical properties, and pharmacokinetic profile. The selected compound was subsequently subjected to molecular docking, ADMET prediction, molecular dynamics simulations, MM-GBSA calculations, DFT analysis, and experimental validation.

Berberine was used as a natural-product lead because of its reported hepatoprotective and metabolic regulatory activity. The molecule was designed as a berberine-inspired analogue rather than a direct one-step derivative of native berberine. The design strategy retained berberine-relevant pharmacophoric characteristics, including an extended aromatic system and heteroatom-rich functionality, while introducing structural features intended to improve FXR binding and drug-like behavior. Specifically, the optimized scaffold incorporated an amide linker, carboxylate functionality, a fluorinated aryl group, and a heteroaryl substituent to enhance hydrogen-bonding potential, electrostatic complementarity, hydrophobic contacts, and lipophilicity within the FXR ligand-binding pocket. These modifications were selected to overcome limitations associated with native berberine, particularly limited target specificity and suboptimal pharmacokinetic behavior.

### 5.1 Docking and interaction analysis

WADDAICA-designed analogues were docked against FXR using PyRx. Binding poses were ranked by binding affinity, and top complexes were analyzed using Discovery Studio Visualizer (v21.1) and SAMSON (v2022) to identify hydrogen bonds, π–π stacking, and hydrophobic interactions.

### 5.2 ADMET analysis

Pharmacokinetic properties were evaluated using SwissADME, including gastrointestinal absorption, BBB permeability, CYP450 interactions, and solubility. Compounds satisfying Lipinski’s rule of five and showing favorable ADMET profiles were prioritized.

### 5.3 Molecular dynamics simulation

Molecular dynamics (MD) simulations were performed to evaluate the structural stability and dynamic behavior of the optimized ligand in complex with the FXR ligand-binding domain.

All simulations were conducted using the Desmond MD engine (Schrödinger Suite, version 2021–4). The initial protein–ligand complex was prepared using the Protein Preparation Wizard in Maestro, which included the addition of hydrogen atoms, assignment of bond orders, optimization of hydrogen-bonding networks, and determination of protonation states at physiological pH (7.4). Missing side chains and loops, if present, were corrected using standard structure refinement protocols.

The prepared complex was embedded in an orthorhombic simulation box with a minimum buffer distance of 10 Å from the protein surface to the box boundaries. The system was solvated using the TIP3P explicit water model. Counterions (Na^+^/Cl^−^) were added to neutralize the system, and an ionic strength of 0.15 M NaCl was maintained to mimic physiological conditions.

Energy minimization was performed using the steepest descent algorithm until convergence, followed by a multi-step equilibration protocol under default Desmond relaxation conditions, including restrained NVT and NPT phases.

Production simulations were carried out under the NPT ensemble at 300 K and 1 atm pressure. Temperature was controlled using the Nose–Hoover thermostat, and pressure was regulated using the Martyna–Tobias–Klein barostat. The OPLS-2005 force field was used for all interactions.

The integration time step was set to 2 fs, and long-range electrostatic interactions were treated using the particle mesh Ewald (PME) method. Trajectories were recorded at intervals of 10 ps over a total simulation time of 500 ns.

Post-simulation analyses included root mean square deviation (RMSD) to evaluate overall structural stability, root mean square fluctuation (RMSF) to assess residue-level flexibility, radius of gyration (Rg) to monitor compactness, and hydrogen bond occupancy to examine ligand–receptor interactions over time. Secondary structure stability was evaluated using trajectory-based analysis.

Binding free energy calculations were performed using the MM-GBSA method implemented in Schrödinger Prime. A total of 100 snapshots were extracted at equal intervals from the final 100 ns of the trajectory to ensure equilibrium sampling. Energy decomposition was used to assess contributions from van der Waals interactions, electrostatics, and solvation effects.

### 5.4 Density functional theory (DFT) analysis

Quantum chemical calculations were conducted to evaluate the electronic properties and chemical stability of the WADDAICA-designed berberine-inspired analogue.

All Density Functional Theory (DFT) calculations were performed using Gaussian 09 W software, and molecular visualization was carried out using GaussView 5.0. The geometry of the WADDAICA-designed analogue was fully refined using the B3LYP functional with the 6–31++G (d,p) basis set.

Geometry optimization was performed in the gas phase without symmetry constraints. Frequency analysis was subsequently conducted to confirm that the optimized structure corresponded to a true energy minimum, indicated by the absence of imaginary frequencies.

Frontier molecular orbitals, including the highest occupied molecular orbital (HOMO) and lowest unoccupied molecular orbital (LUMO), were analyzed to assess electron distribution and reactivity. The HOMO–LUMO energy gap was used as an indicator of molecular stability and chemical reactivity.

Electron density distribution and molecular orbital surfaces were visualized to identify potential regions of electrophilic and nucleophilic interactions relevant to ligand binding within the FXR active site.

## 6 Experimental validation

### 6.1 Synthesis and structural characterization of the WADDAICA-designed berberine-inspired analogue

The WADDAICA-designed berberine-inspired analogue was prepared according to the synthetic route shown in Supplementary Figure S6. The final compound was not treated as a direct structural modification product of native berberine; instead, berberine served as the lead pharmacophoric reference during molecular design. The designed analogue was synthesized through a carbodiimide-mediated amide-coupling strategy using the corresponding carboxylic-acid intermediate and amine-containing fragment.

Briefly, the carboxylic-acid intermediate (100 mg, 1.0 equiv.) was dissolved in anhydrous DMF (5 mL) under nitrogen and cooled to 0 °C. EDC-HCl (1.2 equiv.) and NHS (1.2 equiv.) were added sequentially, and the reaction mixture was stirred at 0 °C for 30 min to activate the carboxylic-acid group and generate the corresponding NHS ester intermediate. Subsequently, the amine-containing fragment (1.1 equiv.) and triethylamine (2.0 equiv.) were added dropwise, and the reaction mixture was allowed to warm to room temperature and stirred for 18 h under nitrogen. Reaction progress was monitored by TLC on silica-gel plates using hexane/ethyl acetate (7:3, v/v) as the mobile phase. TLC was used only to monitor consumption of starting material and appearance of the product spot, not as definitive evidence of molecular identity.

After completion, the reaction mixture was diluted with cold distilled water and extracted with ethyl acetate. The combined organic layers were washed with water and brine, dried over anhydrous sodium sulfate, filtered, and concentrated under reduced pressure. The crude product was purified by silica-gel column chromatography using a hexane/ethyl acetate gradient to afford the target WADDAICA-designed berberine-inspired analogue as a purified solid. The purified compound was dried under vacuum and stored under inert conditions until further use.

The identity and purity of the purified compound were evaluated using ^1^H NMR, ^13^C NMR, LC–MS, HPLC, and FTIR. NMR and LC–MS were used as the principal methods for structural confirmation, whereas HPLC was used to determine chromatographic purity and FTIR was used only as supportive functional-group evidence. HPLC analysis was performed using an Agilent 1260 Infinity system equipped with a reverse-phase C18 column (4.6 × 250 mm, 5 μm). The mobile phase consisted of acetonitrile/water (70:30, v/v) at a flow rate of 1.0 mL/min, with detection at 254 nm and a column temperature of 25 °C. The WADDAICA-designed analogue showed a dominant chromatographic peak at 6.37 min, with chromatographic purity of 98.6%.

FTIR spectra were recorded in the range of 4000–400 cm^-1^ using the KBr pellet method. The FTIR spectrum of the analogue showed characteristic absorptions at 3325 cm^-1^, assigned to N–H stretching, 1652 cm^-1^, assigned to amide C=O stretching, 1522 cm^-1^, assigned to aromatic C=C stretching, 1278 cm^-1^, assigned to C–O–C stretching, and 1105 cm^-1^, assigned to C–F stretching. The absence of a broad free carboxylic-acid O–H band in the 3300–2500 cm^-1^ region was consistent with consumption of the carboxylic-acid intermediate during amide-bond formation. Because amide carbonyl vibrations may overlap with conjugated aromatic or heteroaromatic absorptions, FTIR was not used as the sole method for structural assignment.

The ^1^H NMR and ^13^C NMR spectra showed signals consistent with the expected aromatic, heteroaromatic, linker-associated, and carbonyl-associated environments of the proposed structure. LC–MS analysis showed the expected molecular ion corresponding to the calculated molecular mass of the designed analogue. Together, the NMR, LC–MS, HPLC, and FTIR results supported successful synthesis, purification, and structural confirmation of the WADDAICA-designed berberine-inspired analogue. For *in vitro* experiments, the purified compound was dissolved in DMSO to prepare a stock solution and diluted freshly in culture medium before treatment. The final DMSO concentration was maintained at ≤0.1% in all treated and vehicle-control groups. Cells were treated with native berberine, the WADDAICA-designed analogue, or CDCA at concentrations ranging from 1 to 100 μM for 24 and 48 h.

### 6.2 Cell culture

Human hepatocellular carcinoma HepG2 cells (ATCC HB-8065) were obtained from the American Type Culture Collection. Cells were cultured in Dulbecco’s Modified Eagle Medium (DMEM, high glucose) supplemented with 10% fetal bovine serum and 1% penicillin–streptomycin.

Cells were maintained at 37 °C in a humidified incubator with 5% CO_2_. Sub culturing was performed at 80%–90% confluence using 0.25% trypsin–EDTA. Cells between passages 5 and 20 were used for all experiments. Routine *mycoplasma* testing was conducted using PCR-based detection methods.

### 6.3 Induction of MASH-like cellular model

A MASH-like phenotype was induced by treating HepG2 cells with a free fatty acid (FFA) mixture consisting of oleic acid and palmitic acid in a 2:1 M ratio. The FFA mixture was conjugated to fatty acid-free bovine serum albumin (BSA) at a 6:1 M ratio and applied at a final concentration of 0.5 mM.

Cells were incubated with the FFA mixture for 24–48 h to induce lipid accumulation, oxidative stress, and inflammatory signaling characteristic of steatotic liver conditions. This model was used as a preliminary *in vitro* MASH-like system for evaluating steatosis-associated cellular stress and metabolic dysfunction. However, it does not fully reproduce the multicellular inflammatory, fibrotic, and immunological complexity of human MASH.

### 6.4 MTT cytotoxicity assay

Cell viability was assessed using the MTT assay. HepG2 cells were seeded in 96-well plates at a density of 1 × 10^4^ cells per well and allowed to adhere overnight.

Cells were treated with native berberine, berberine-inspired analogue, or CDCA at concentrations ranging from 1 to 100 µM for 24 and 48 h. Following treatment, MTT reagent (0.5 mg/mL in PBS) was added and incubated for 4 h at 37 °C.

The medium was removed, and formazan crystals were dissolved in dimethyl sulfoxide (DMSO). Absorbance was measured at 570 nm with a reference wavelength of 630 nm using a BioTek Synergy HTX microplate reader. Results were expressed as a percentage of untreated control.

### 6.5 Lipid accumulation assay

Cells were cultured on coverslips and subjected to FFA-induced MASH conditions followed by treatment with test compounds. Cells were fixed with 4% paraformaldehyde for 15 min and permeabilized with 0.1% Triton X-100.

Neutral lipid accumulation was visualized using BODIPY 493/503 dye at a concentration of 1 μg/mL for 30 min. Nuclei were counterstained with DAPI (1 μg/mL, 5 min).

Images were acquired using a Zeiss LSM 880 confocal microscope, and fluorescence intensity was quantified using ImageJ software.

### 6.6 Immunocytochemistry

Cells were fixed and blocked with 5% bovine serum albumin for 1 h. Primary antibodies against FXR, SREBP-1c, TNF-α, and α-SMA were applied at dilutions ranging from 1:200 to 1:500 and incubated overnight at 4 °C.

After washing, cells were incubated with Alexa Fluor-conjugated secondary antibodies (1:1000) for 1 h at room temperature in the dark. Nuclei were stained with DAPI, and images were captured using a fluorescence microscope. Quantification was performed using ImageJ.

### 6.7 Western blot analysis

Total protein was extracted using RIPA buffer containing protease inhibitors. Protein concentration was determined using the BCA assay.

Equal amounts of protein (30–50 µg) were separated on 10%–12% SDS-PAGE gels and transferred onto PVDF membranes. Membranes were blocked with 5% non-fat milk and incubated overnight at 4 °C with primary antibodies against FXR, PPARα, SREBP-1c, TNF-α, IL-6, α-SMA, collagen I, and cleaved caspase-3 at a dilution of 1:1000.

Membranes were then incubated with HRP-conjugated secondary antibodies (1:5000) for 1 h at room temperature. Bands were visualized using enhanced chemiluminescence and imaged using a Bio-Rad ChemiDoc system. Densitometric analysis was performed using ImageJ, with β-actin used as the loading control.

### 6.8 Quantitative real-time PCR

Total RNA was extracted using TRIzol reagent, and purity was assessed using a NanoDrop spectrophotometer. cDNA was synthesized from 1 µg RNA using a reverse transcription kit.

qPCR was performed using SYBR Green master mix on an Applied Biosystems QuantStudio real-time PCR system. Target genes included FXR, SREBP1, PPARA, TNFA, IL6, COL1A1, ACTA2, and CYP7A1, with GAPDH used as an internal control.

Relative gene expression was calculated using the 2^(−ΔΔCt)^ method. Cycling conditions included initial denaturation at 95 °C for 5 min followed by 40 cycles of amplification.

### 6.9 Statistical analysis

All cell-based experiments were performed using three independent biological replicates. Each biological replicate represented an independently cultured, treated, harvested, and analyzed cell preparation. When technical measurements were obtained within the same biological experiment, these technical values were first averaged to generate one value for that biological replicate. Statistical analysis was then performed using only the biological-replicate values.

For immunocytochemistry, fluorescence intensity was quantified from randomly selected microscopic fields under identical acquisition settings and normalized to the control group. For Western blot analysis, densitometric values of target proteins were normalized to β-actin within the same blot before fold-change calculation. For qPCR analysis, gene expression was normalized to GAPDH and calculated using the 2^−ΔΔCT^ method. Mean and standard deviation were calculated from the normalized biological-replicate values. SD values were not calculated from pooled technical measurements, raw band intensities, raw fluorescence values, or raw Ct values.

Statistical analysis was performed using GraphPad Prism version 9.0. Differences among groups were evaluated using one-way ANOVA followed by Tukey’s multiple-comparison *post hoc* test. Statistical significance was defined as p < 0.05.

### 6.10 Biosafety compliance

All experiments involving human-derived cell lines were conducted in a certified biosafety level 2 (BSL-2) laboratory in accordance with institutional biosafety guidelines.

## 7 Results

### 7.1 Performance of SE attention-based transformer for liver disease prediction

**FIGURE 3:**
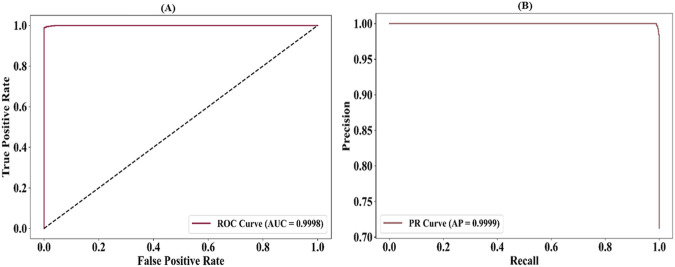
Performance evaluation of the SE attention-based Transformer model for liver disease classification. **(A)** Receiver operating characteristic (ROC) curve showing classification performance on the independent test dataset, with an area under the curve (AUC) of 0.9998. **(B)** Precision–recall (PR) curve with an average precision (AP) of 0.9999. The model was trained using stratified data partitioning and evaluated on the held-out test set. AUC, area under the curve; AP, average precision.

The SE attention-based Transformer model demonstrated excellent predictive performance for liver disease classification. The receiver operating characteristic (ROC) curve exhibited near-perfect discrimination with an area under the curve (AUC) of 0.9998, indicating high sensitivity and specificity across classification thresholds (Figure 3A). Complementary precision–recall analysis further confirmed robust model performance under class imbalance conditions, with an average precision (AP) of 0.9999 (Figure 3B).

**FIGURE 4:**
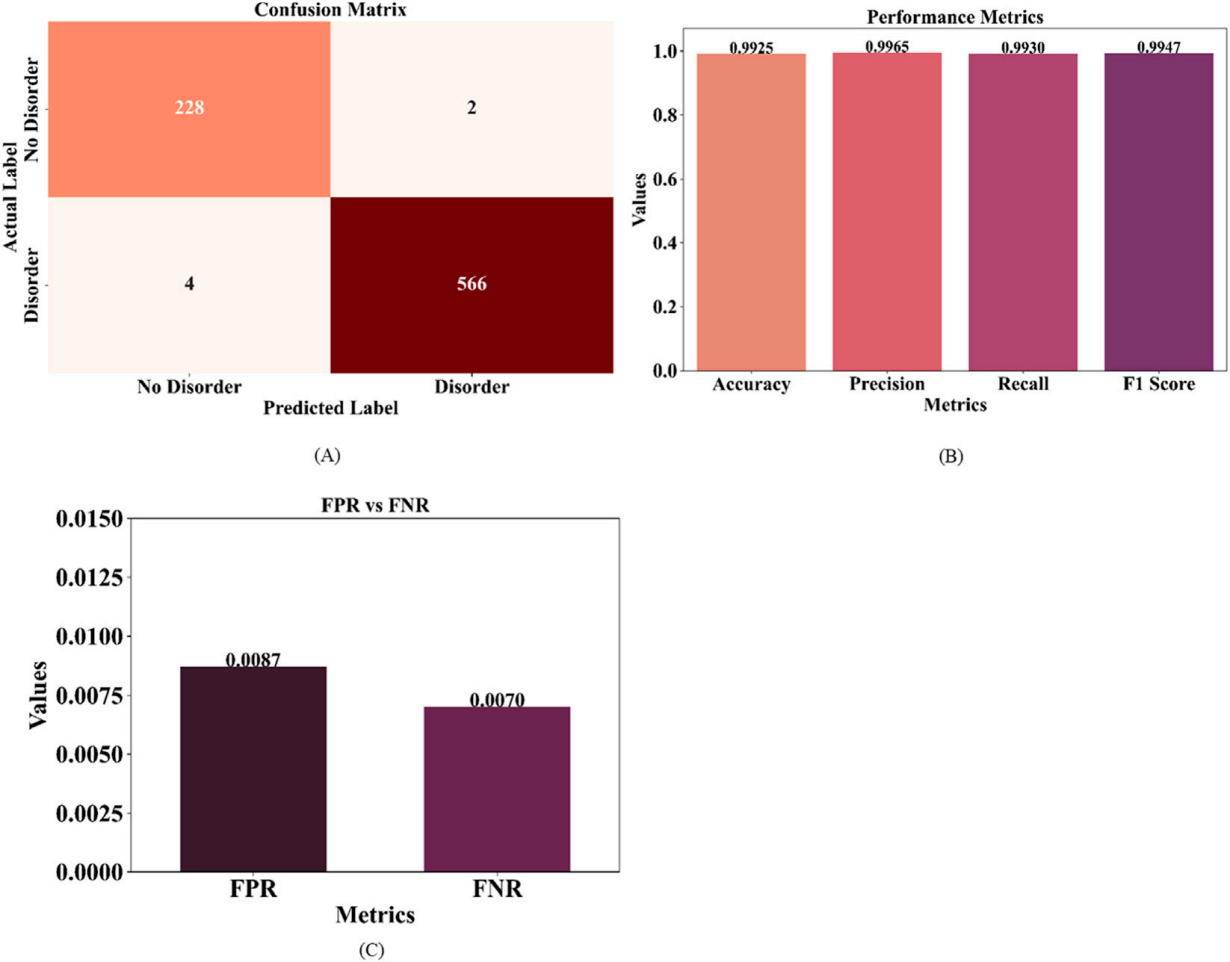
Classification performance of the SE attention-based Transformer model for liver disease prediction **(A)** Confusion matrix summarizing classification outcomes on the test dataset. The model correctly identifies the majority of cases, with 566 true positives and 228 true negatives, and minimal misclassification (false positives = 2; false negatives = 4), indicating high predictive accuracy. **(B)** Quantitative performance metrics of the model, including accuracy (0.9925), precision (0.9965), recall (0.9930), and F1-score (0.9947), demonstrating balanced classification performance across evaluation criteria. **(C)** Comparison of false positive rate (FPR) and false negative rate (FNR), showing low error rates (FPR = 0.0087; FNR = 0.0070), indicating reliable discrimination between disease and non-disease classes.

Classification performance was further validated using a confusion matrix, which showed a high number of true positive and true negative predictions with minimal misclassification (Figure 4A). Quantitative performance metrics, including accuracy (0.9925), precision (0.9965), recall (0.9930), and F1-score (0.9947), confirmed balanced and reliable classification (Figure 4B). Additionally, the model exhibited very low diagnostic error rates, as indicated by low false positive rate (FPR = 0.0087) and false negative rate (FNR = 0.0070) (Figure 4C).

Training dynamics showed stable convergence, with progressive increases in training and validation accuracy and corresponding reductions in loss, without evidence of overfitting (Supplementary Figures S1A,B). The high performance is attributed to structured clinical features and controlled training strategies, including stratified data splitting and early stopping.

**FIGURE 5:**
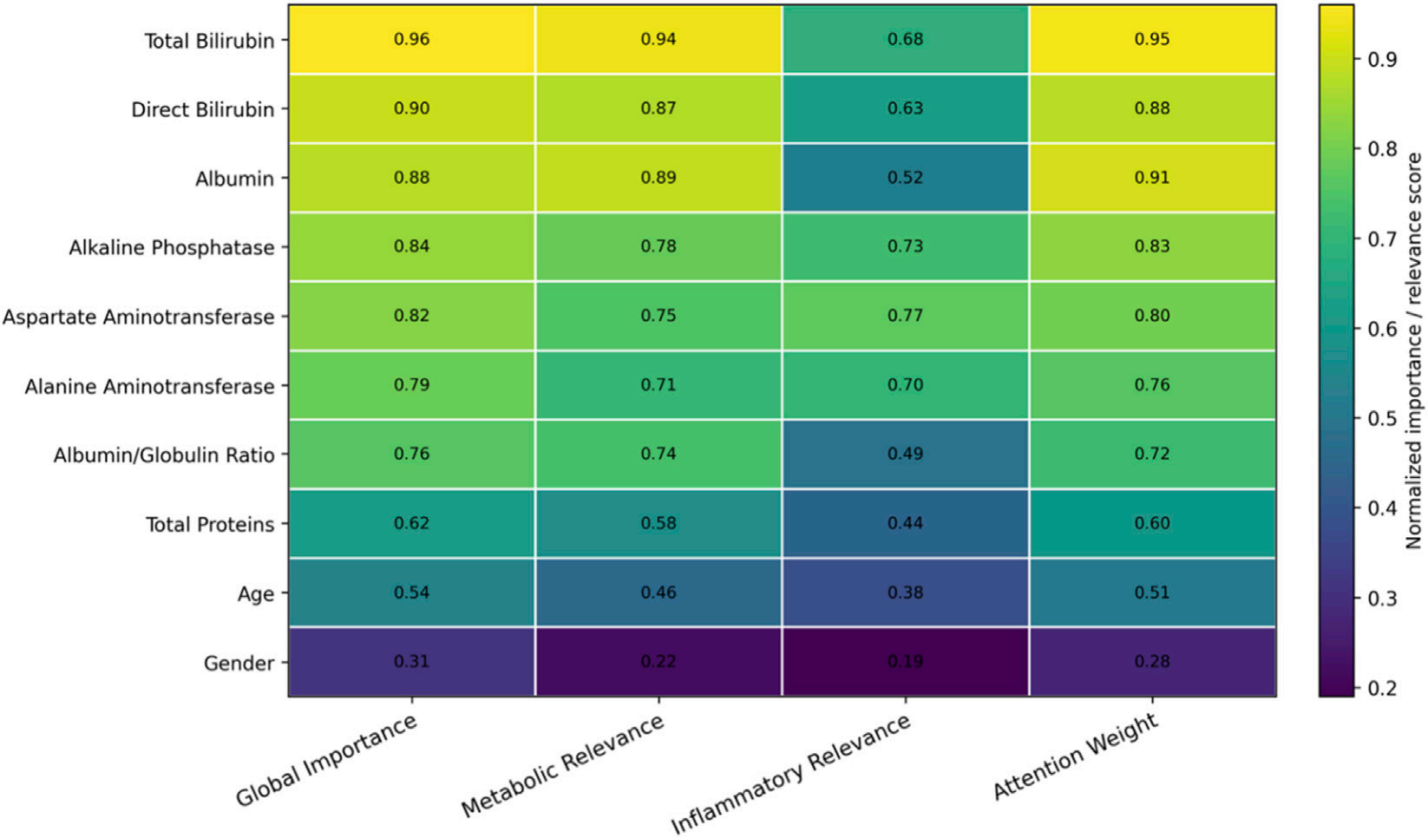
Feature importance heatmap of clinical predictors contributing to SE attention-based liver disease classification. Heatmap showing normalized relative importance of major clinical variables used in the SE attention-based Transformer model. The matrix was organized to reflect overall model contribution, metabolic relevance, inflammatory relevance, and attention prioritization across core liver-associated biomarkers. Bilirubin fractions, albumin, and liver enzyme measures showed the strongest relative contribution, consistent with their biological relevance to hepatic dysfunction and FXR-linked metabolic regulation.

Feature importance analysis revealed that bilirubin fractions, albumin, and liver enzyme markers contributed most significantly to model predictions (Figure 5). These features are well-established components of metabolic liver dysfunction and are closely associated with FXR-regulated pathways, supporting the biological relevance of model-derived insights.

### 7.2 Biological identification of FXR as a central regulatory target

To translate AI-derived clinical insights into biological mechanisms, FXR (NR1H4) was selected as a key regulatory target due to its established role in bile acid metabolism, lipid homeostasis, and inflammatory signaling.

**FIGURE 6:**
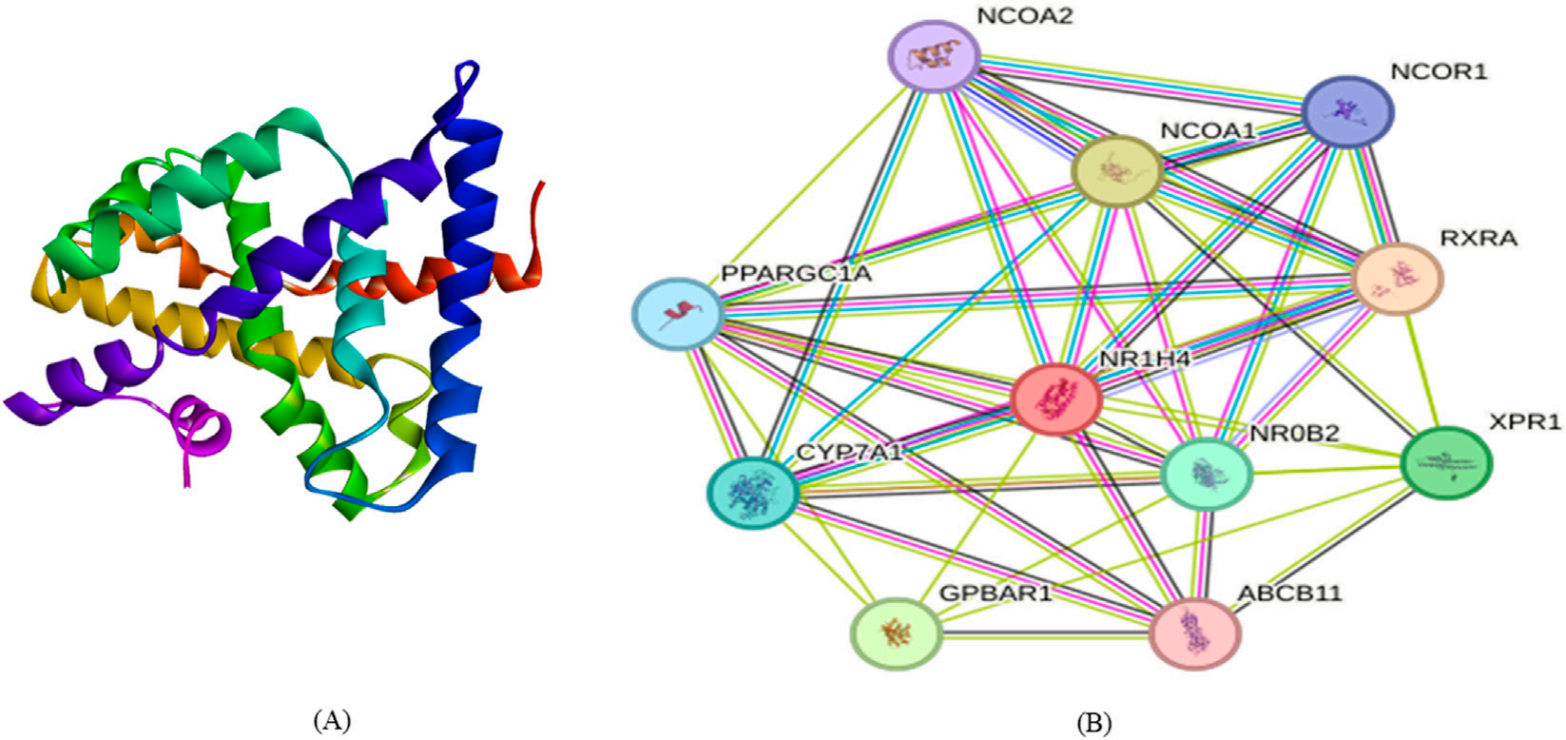
Structural and network characterization of FXR (NR1H4) as a therapeutic target in liver disease **(A)** Three-dimensional crystal structure of the farnesoid X receptor ligand-binding domain (FXR-LBD) in complex with a co-crystallized ligand (HNC143) and coactivator SRC1 (PDB ID: 6A5W). The structure was obtained from the Protein Data Bank and used for molecular docking and structure-based drug design. **(B)** Protein–protein interaction (PPI) network of FXR (NR1H4) generated using the STRING database (confidence score ≥ 0.7), illustrating key interacting partners involved in bile acid metabolism (CYP7A1), lipid regulation (PPARGC1A), nuclear receptor signaling (RXRA, NR0B2), and hepatic transport processes (ABCB11). The network highlights the central regulatory role of FXR in hepatic metabolic and signaling pathways.

Structural analysis of the FXR ligand-binding domain (LBD) confirmed a well-defined binding pocket suitable for ligand interaction (Figure 6A). Protein–protein interaction (PPI) network analysis revealed that FXR is centrally connected to key metabolic regulators, including CYP7A1, RXRA, and PPARGC1A, highlighting its role in hepatic metabolic regulation (Figure 6B).

**FIGURE 7:**
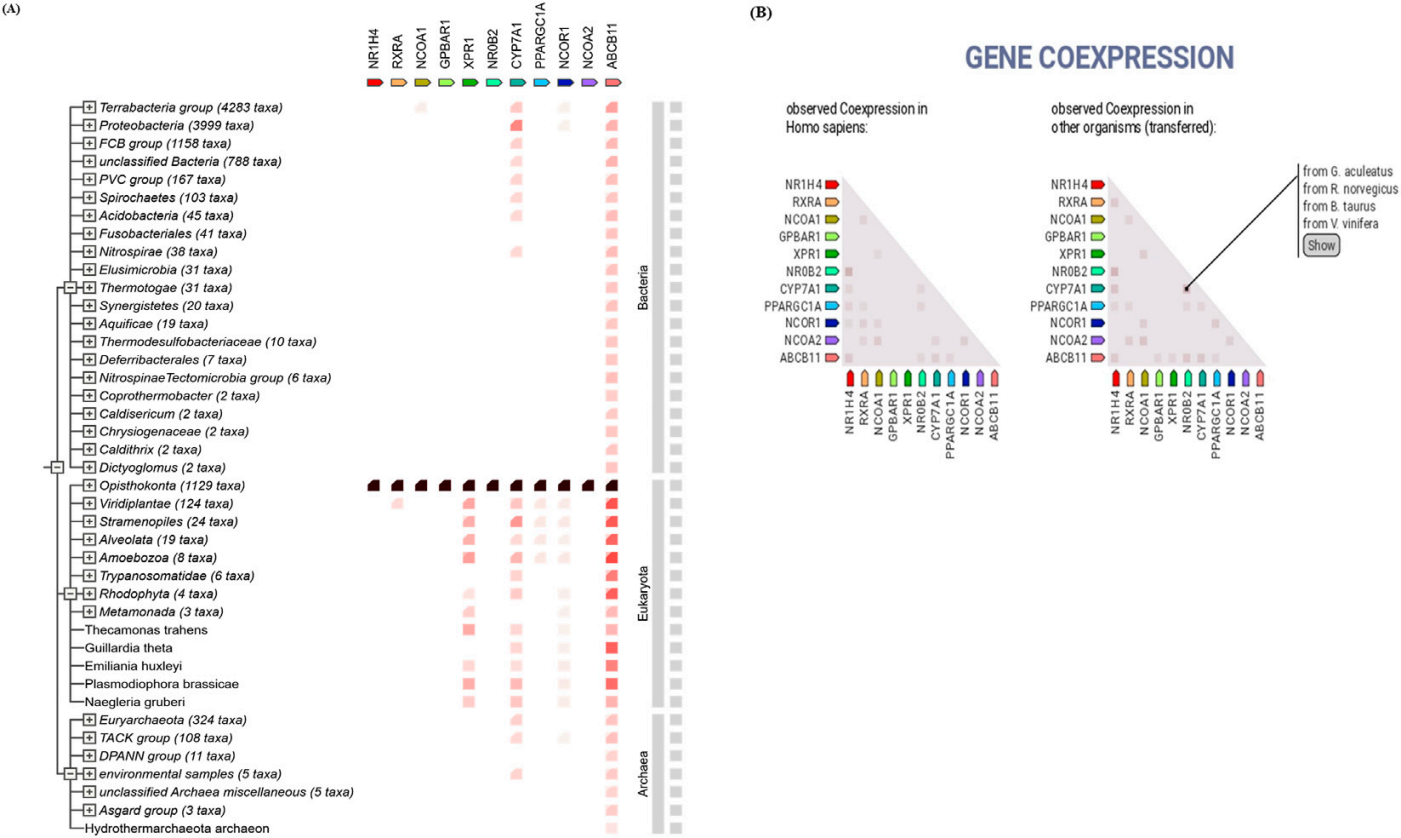
Cross-species conservation and co-expression patterns of FXR (NR1H4)-associated regulatory genes **(A)** Heatmap and hierarchical clustering of FXR-associated genes across multiple taxa, illustrating conserved patterns of gene enrichment in metabolic and regulatory pathways. Genes involved in bile acid metabolism (CYP7A1), nuclear receptor signaling (RXRA, NR0B2), and transcriptional regulation (PPARGC1A, NCOA1/2) show coordinated clustering, supporting the conserved functional role of FXR across biological systems. **(B)** Gene co-expression analysis of FXR (NR1H4) and associated regulatory genes in *Homo sapiens* and across other organisms. The co-expression patterns demonstrate strong correlation among FXR-related genes, including RXRA, CYP7A1, and PPARGC1A, indicating conserved regulatory networks involved in lipid metabolism, bile acid homeostasis, and hepatic function.

Cross-species conservation and co-expression analysis further demonstrated that FXR-associated genes exhibit coordinated expression patterns across multiple biological systems (Figures 7A,B). The observed clustering of genes involved in bile acid metabolism, nuclear receptor signaling, and transcriptional regulation supports the conserved functional importance of FXR in metabolic homeostasis.

### 7.3 Virtual screening and identification of lead compounds targeting FXR

Binding site prediction using PrankWeb identified three potential ligand-binding pockets within the FXR LBD, with Pocket one exhibiting the highest confidence score and structural accessibility (Table 1). This pocket was selected for subsequent docking studies.

**TABLE 1:** High-confidence ligand-binding pockets of the FXR ligand-binding domain identified using PrankWeb analysis.

Pocket ID	Rank	Score	Probability	SAS points	Surface atoms	Center coordinates (x, y, z) (Å)
Pocket 1	1	37.33	0.949	135	67	(13.77, −14.07, −14.22)
Pocket 2	2	1.80	0.033	27	13	(11.80, −27.76, −27.90)
Pocket 3	3	1.06	0.008	20	11	(11.10, −10.32, −34.66)

• Predicted ligand-binding pockets within the farnesoid X receptor (FXR) ligand-binding domain (PDB ID: 6A5W) were identified using the PrankWeb server. Pocket ranking is based on prediction score and probability, reflecting the likelihood of biologically relevant ligand interaction. Pocket 1 exhibited the highest confidence and was selected for subsequent molecular docking and simulation studies, consistent with its large solvent-accessible surface and favorable spatial positioning within the FXR active site.

• Score: Composite metric generated by PrankWeb reflecting pocket quality and druggability.

• Probability: Likelihood of the predicted pocket being a true ligand-binding site.

• SAS Points: Number of solvent-accessible surface points contributing to pocket definition.

• Surface Atoms: Number of protein atoms forming the pocket boundary.

• Center Coordinates (x, y, z) (Å): Geometric center of the predicted binding pocket used for docking grid definition.

• Coordinates are reported in angstroms (Å).

A library of natural compounds was screened for FXR binding potential, including reference agonists chenodeoxycholic acid (CDCA) and obeticholic acid (OCA) (Table 2; Supplementary Table S1). Molecular docking analysis predicted favorable binding interactions between several screened compounds and the FXR ligand-binding pocket. CDCA exhibited the most favorable docking score, while berberine demonstrated a docking profile comparable to known FXR modulators. These results supported the selection of berberine as a lead candidate for further computational and experimental investigation (Table 3; Supplementary Table S2).

**TABLE 2:** Representative natural compounds screened for FXR targeting, including reference agonists.

Compound	Class	PubChem CID	Role
Berberine	Isoquinoline alkaloid	2353	Selected lead compound
Curcumin	Polyphenol	969516	Reference natural compound
Genistein	Isoflavone	5280961	Representative flavonoid
Piperine	Alkaloid	638024	Bioavailability enhancer
Rutin	Flavonoid glycoside	5280805	Polyphenolic compound
EGCG	Catechin	65064	Antioxidant compound
Chenodeoxycholic acid (CDCA)	Bile acid	10133	Endogenous FXR ligand
Obeticholic acid (OCA)	Bile acid derivative	447715	Clinical putative FXR modulator

**TABLE 3:** Docking affinity and drug-likeness evaluation of top compounds predicted to interact with FXR-associated pathways identified through virtual screening.

Compound	Binding energy (kcal/mol)	Solubility	GI absorption	BBB	Lipinski	Toxicity
Chenodeoxycholic acid (CDCA)	−10.8	Soluble	High	No	✔ (0)	Class 4
Berberine	−9.2	Moderate	High	Yes	✔ (0)	Class 3
Obeticholic acid (OCA)	−9.0	Moderate	High	No	✔ (1)	Class 4
Curcumin	−8.7	Soluble	High	No	✔ (0)	Class 4
Genistein	−8.5	Soluble	High	No	✔ (0)	Class 5
Piperine	−8.5	Soluble	High	Yes	✔ (0)	Class 4
Rutin	−8.5	Soluble	Low	No	✘ (3)	Class 5
EGCG	−8.4	Soluble	Low	No	✘ (2)	Class 4

• Molecular docking and in silico pharmacokinetic profiling of selected natural compounds targeting the farnesoid X receptor (FXR). Binding affinity was calculated using AutoDock Vina, and drug-likeness properties were evaluated using SwissADME and ProTox-II. Chenodeoxycholic acid (CDCA) and obeticholic acid (OCA) were included as reference FXR modulators. Berberine demonstrated favorable binding affinity and pharmacokinetic properties, supporting its selection for further structural optimization.

• Binding Energy: Predicted binding affinity from molecular docking (AutoDock Vina); more negative values indicate stronger binding.

• Solubility: Predicted aqueous solubility (ESOL classification).

• GI Absorption: Predicted gastrointestinal absorption (High/Low) from SwissADME.

• BBB: Blood–brain barrier permeability prediction (Yes/No).

• Lipinski: Drug-likeness based on Lipinski’s Rule of Five; values in parentheses indicate number of violations.

• Toxicity Class: Predicted oral toxicity class (ProTox-II; Class 1 = most toxic, Class 6 = least toxic).

• EGCG: Epigallocatechin gallate.

• ✔ indicates compliance; ✘ indicates violation.

In addition to favorable binding affinity, berberine showed favorable pharmacokinetic properties, including high gastrointestinal absorption and compliance with Lipinski’s rule of five (Table 3), supporting its selection as a lead compound for further optimization.

### 7.4 WADDAICA-guided design and physicochemical characterization of the berberine-inspired analogue

After clinical AI-assisted feature learning and FXR prioritization, molecular design was performed independently using WADDAICA. The diagnostic Transformer model was not used to optimize the compound. The resulting WADDAICA-designed berberine-inspired analogue exhibited favorable physicochemical properties, including moderate molecular weight, balanced lipophilicity (consensus LogP = 2.83), and appropriate polarity (TPSA = 102.68 Å^2^), supporting drug-likeness and membrane permeability (Table 4).

**TABLE 4:** Structural and physicochemical characterization of the WADDAICA-designed berberine-inspired analogue targeting FXR.

Category	Parameter	Value
Structure	SMILES	COc1cc (C (=O)NC(CC(=O)O)c2ccc(F)c(F)c2)ccc1OCc1cnn(C)c1
Molecular identity	Molecular formula	C_22_H_21_F_2_N_3_O_5_
	Molecular weight (g/mol)	445.42
Molecular properties	Heavy atoms	32
	Aromatic atoms	17
	Fraction Csp^3^	0.23
	Rotatable bonds	10
	H-bond acceptors/Donors	8/2
Polarity and size	TPSA (Å^2^)	102.68
	Molar refractivity	109.74
Lipophilicity	Consensus LogP	2.83
	LogP range (min–max)	2.17 – 3.60

• Physicochemical and structural properties of the WADDAICA-designed analogue inspired analogue designed for enhanced interaction with the farnesoid X receptor (FXR). The compound exhibits moderate molecular weight, balanced lipophilicity, and suitable polarity, supporting membrane permeability and receptor binding. These properties indicate favorable drug-like characteristics for targeting FXR-mediated metabolic pathways.

• SMILES: Simplified molecular-input line-entry system representation of the compound.

• TPSA: Topological polar surface area, indicative of permeability and absorption.

• LogP: Octanol–water partition coefficient; consensus value derived from multiple prediction models.

• LogP Range: Variation across computational methods (iLOGP, XLOGP3, WLOGP, MLOGP, SILICOS-IT).

• All properties were predicted using SwissADME.

Pharmacokinetic analysis predicted high gastrointestinal absorption, low blood–brain barrier permeability, and favorable drug-likeness profiles with no PAINS or Brenk alerts (Table 5). These findings indicate that the WADDAICA-designed analogue retains desirable pharmacological properties while improving target specificity.

**TABLE 5:** Predicted pharmacokinetic, drug-likeness, and medicinal chemistry profile of the WADDAICA-designed berberine-inspired analogue (SwissADME analysis).

Category	Parameter	Value
Solubility	LogS (ESOL)	−3.70
	LogS (ali)	−3.96
	LogS (SILICOS-IT)	−6.37
	Solubility class	Soluble to moderately soluble
Pharmacokinetics	GI absorption	High
	BBB permeability	No
	P-gp substrate	Yes
	CYP inhibition	CYP2C19, CYP2C9, CYP2D6, CYP3A4
	Skin permeation (log Kp, cm/s)	−7.48
Drug-likeness	Lipinski rule	✔ (0 violations)
	Ghose/Veber/Egan/Muegge	✔ Compliant
	Bioavailability score	0.56
Medicinal chemistry	PAINS alerts	0
	Brenk alerts	0
	Lead-likeness	✘ (2 violations: MW > 350, rotatable bonds > 7)
	Synthetic accessibility	3.41

• In silico pharmacokinetic and drug-likeness evaluation of the WADDAICA-designed berberine-inspired analogue targeting the farnesoid X receptor (FXR). The compound demonstrates high predicted gastrointestinal absorption, acceptable solubility, and favorable drug-likeness profiles with no PAINS or Brenk alerts. Moderate lipophilicity and controlled polarity support membrane permeability, while predicted CYP450 interactions suggest the need for further metabolic optimization. Overall, the profile supports its suitability for FXR-targeted therapeutic development.

• LogS: Predicted aqueous solubility using ESOL, Ali, and SILICOS-IT models.

• GI Absorption: Predicted intestinal absorption (SwissADME).

• BBB: Blood–brain barrier permeability prediction.

• P-gp: P-glycoprotein substrate prediction, indicating efflux potential.

• CYP Inhibition: Predicted inhibition of cytochrome P450 isoforms relevant to drug metabolism.

• Lipinski Rule: Drug-likeness based on Rule of Five; ✔ indicates compliance.

• PAINS: Pan-assay interference compounds alerts.

• Brenk Alerts: Structural alerts for potential toxicity or instability.

• Synthetic Accessibility: Score ranging from one (easy) to 10 (difficult).

• Predictions were performed using SwissADME.

HPLC was used to evaluate chromatographic purity and retention behavior, TLC was used to monitor reaction progress, and FTIR was used only to support functional-group assignment. Molecular identity was assigned primarily using ^1^H NMR, ^13^C NMR, and LC–MS data (Supplementary Table S3; Supplementary Figure S3B).

### 7.5 Structural confirmation of the WADDAICA-designed berberine-inspired analogue

The WADDAICA-designed berberine-inspired analogue was characterized using complementary analytical methods. ^1^H NMR and ^13^C NMR were used as the principal spectroscopic methods for assigning the expected proton and carbon environments of the proposed structure, while LC–MS was used to verify the molecular ion corresponding to the designed analogue. HPLC analysis demonstrated chromatographic purity but was not used as independent proof of molecular identity. FTIR analysis was used only as supportive functional-group evidence.

The FTIR spectrum showed absorptions consistent with N–H stretching, amide-associated C=O vibration, aromatic C=C stretching, C–O–C stretching, and C–F stretching. The absence of a broad free carboxylic-acid O–H band was consistent with consumption of the carboxylic-acid intermediate during amide-bond formation. However, because FTIR bands can overlap in conjugated aromatic and heteroaromatic systems, FTIR was not used as the sole method for structure elucidation. Similarly, TLC and HPLC retention behavior were interpreted only as evidence of chromatographic difference and purity, not as definitive confirmation of structure.

Detailed spectral assignments obtained from ^1^H NMR and ^13^C NMR analyses are summarized in Supplementary Tables S5, S6, while the corresponding spectra are presented in Supplementary Figures S7, S8. LC–MS molecular ion and fragment assignments are provided in Supplementary Table S7 and Supplementary Figure S9, and HPLC chromatographic purity data are presented in Supplementary Table S8 and Supplementary Figure S10.

Collectively, the NMR, LC–MS, and HPLC data support the proposed structure and high purity of the synthesized berberine-inspired analogue.

### 7.6 Binding mode and interaction analysis with FXR

**FIGURE 8:**
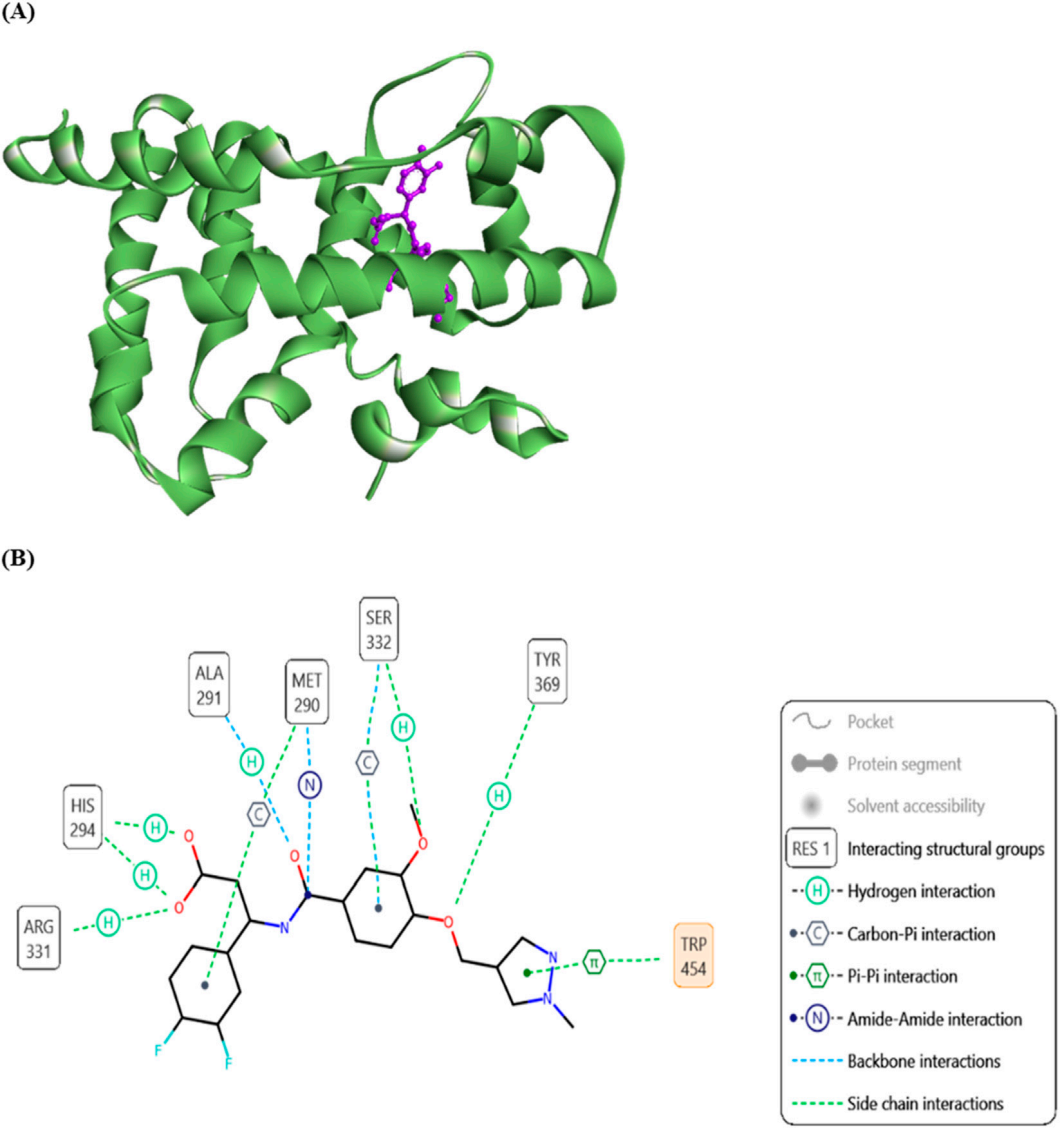
Predicted binding mode of the WADDAICA-designed berberine-inspired analogue within the FXR ligand-binding domain. **(A)** Three-dimensional representation of the top-ranked docking pose generated using AutoDock Vina. **(B)** Two-dimensional interaction diagram illustrating predicted hydrogen-bonding, hydrophobic, and π-interactions with key amino acid residues within the binding pocket. The docking results represent computational predictions of potential ligand–receptor interactions and do not constitute direct experimental evidence of binding.

Docking analysis predicted a favorable binding pose of the WADDAICA-designed analogue within the FXR ligand-binding pocket. The modeled ligand–receptor complex showed multiple potential interactions involving residues such as MET290, SER332, TYR369, and ARG331, supporting the possibility of productive receptor engagement (Figure 8A). The ligand formed key interactions with residues such as MET290, SER332, TYR369, and ARG331, including hydrogen bonds, π–π stacking, and hydrophobic contacts (Figure 8B). These interactions are consistent with known Putative FXR modulator binding modes and support effective ligand–receptor engagement. Previous structural studies of FXR agonists have shown that residues including ARG331, SER332, MET290, TYR358, HIS447, and TYR369 play important roles in ligand recognition and receptor activation. In particular, the clinically approved FXR agonist obeticholic acid (OCA) and the endogenous ligand chenodeoxycholic acid (CDCA) have been reported to establish stabilizing interactions with residues located within the same ligand-binding cavity. Notably, the WADDAICA-designed berberine-inspired analogue retained predicted interactions with ARG331, SER332, MET290, and TYR369, suggesting partial overlap with the binding environment utilized by established FXR ligands (Downes et al., 2003; Mi et al., 2003). Although the docking results do not establish direct agonist activity, the observed interaction pattern supports the possibility that the optimized analogue occupies a biologically relevant region of the FXR ligand-binding pocket.

### 7.7 Molecular dynamics simulation supports potential stability of the FXR–ligand complex

**FIGURE 9:**
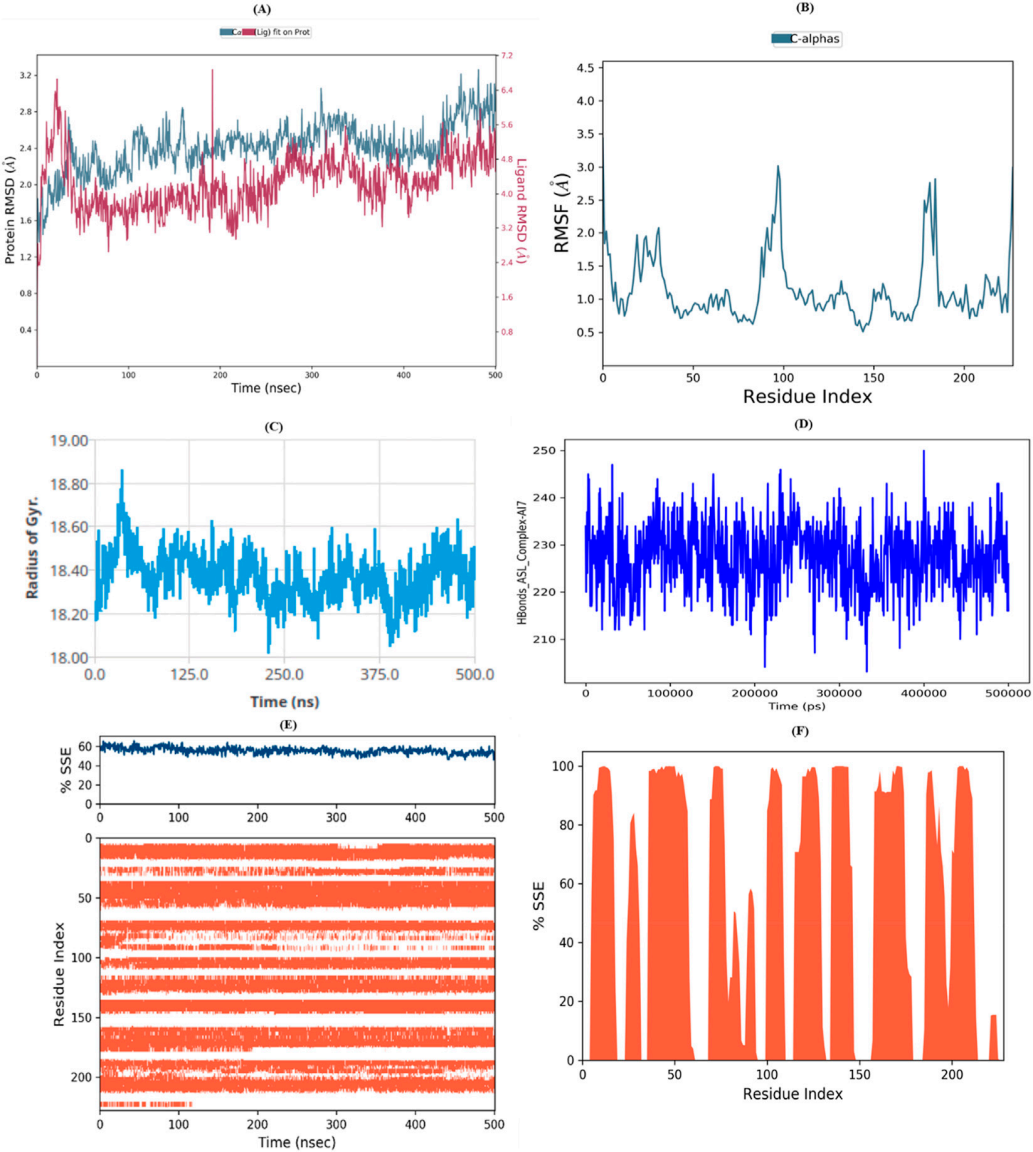
Molecular dynamics analysis of the predicted FXR–ligand complex during a 500 ns simulation. **(A)** RMSD of the protein–ligand complex. **(B)** RMSF of individual protein residues. **(C)** Radius of gyration (Rg). **(D)** Hydrogen bond occupancy. **(E,F)** Secondary structure evolution during simulation. These analyses provide computational evidence supporting potential stability of the predicted complex under simulated conditions.

Molecular dynamics (MD) simulations over 500 ns demonstrated stable behavior of the FXR–ligand complex. Root mean square deviation (RMSD) analysis showed initial equilibration followed by stable convergence, indicating structural stability (Figure 9A). Residue-level flexibility analysis (RMSF) revealed minimal fluctuations in key binding-site regions, with flexibility primarily confined to loop regions (Figure 9B).

The radius of gyration remained stable throughout the simulation, confirming maintenance of structural compactness (Figure 9C). Persistent hydrogen bond formation between the ligand and receptor further supported stable binding interactions (Figure 9D). Secondary structure analysis indicated preservation of protein architecture during the simulation (Figures 9E,F).

Additional MD parameters, including solvent-accessible surface area and system energy, remained stable over time, indicating thermodynamic stability of the complex (Supplementary Figures S2A,B).

Binding free energy analysis using MM-GBSA revealed improved binding affinity after simulation (ΔG_bind = −125.07 kcal/mol at 500 ns), driven primarily by electrostatic and lipophilic interactions (Table 6). These findings support the possibility of a stable ligand–receptor interaction during the simulation period and provide computational evidence consistent with sustained binding within the FXR binding pocket.

**TABLE 6:** Binding free energy and key energetic contributions of the WADDAICA-designed analogue–FXR complex derived from MM-GBSA analysis.

Parameter	0 ns	500 ns
ΔG_bind (kcal/mol)	−118.18	−125.07
Electrostatic (Coulomb)	−13.44	−27.51
Hydrogen bonding	−0.68	−1.97
Lipophilic interactions	−77.75	−71.41
Solvation energy (GB)	30.99	29.42

• Predicted binding free energies of the WADDAICA-designed berberine-inspired analogue in complex with FXR estimated using the MM-GBSA method. These values provide computational estimates of relative binding favorability and should not be interpreted as direct experimental measurements of binding affinity.

• ΔG_bind: Total binding free energy estimated using the MM-GBSA method.

• Electrostatic (Coulomb): Contribution from electrostatic interactions between ligand and receptor.

• Hydrogen Bonding: Stabilization arising from hydrogen bond formation.

• Lipophilic Interactions: Hydrophobic contributions to binding stability.

• Solvation Energy (GB): Generalized Born solvation energy component.

• Values are averaged from representative frames extracted from molecular dynamics simulations.

• Calculations were performed using Schrödinger Prime MM-GBSA module.

### 7.8 Electronic structure analysis provides insight into molecular reactivity and electronic properties

**FIGURE 10:**
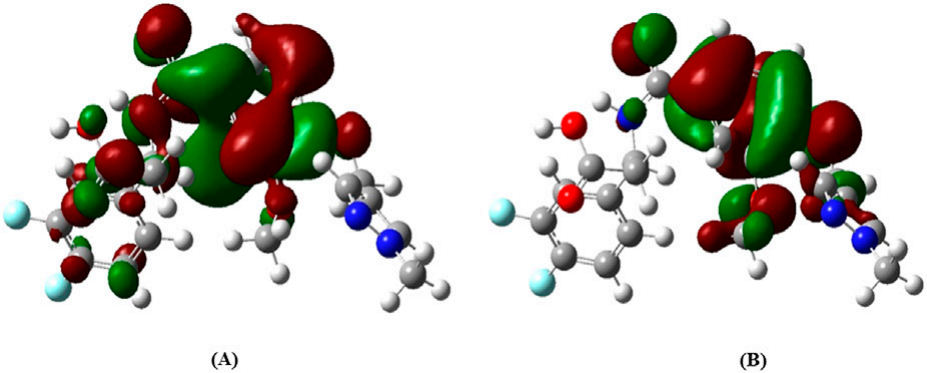
Density functional theory (DFT) analysis of the WADDAICA-designed berberine-inspired analogue. **(A)** HOMO distribution. **(B)** LUMO distribution. The calculated frontier molecular orbitals illustrate electron density distribution and provide information regarding molecular reactivity and electronic properties. DFT analysis is presented as supplementary physicochemical characterization and does not directly assess FXR binding or biological activity.

Density functional theory (DFT) analysis revealed favorable electronic properties of WADDAICA-designed berberine-inspired analogue. The spatial distribution of HOMO and LUMO orbitals indicated distinct regions of electron donation and acceptance (Figures 10A,B), supporting balanced reactivity.

These electronic properties provide complementary information regarding molecular reactivity and charge distribution. While such characteristics may influence ligand–receptor interactions, DFT analysis does not directly assess FXR binding or biological activity and therefore should be interpreted as supportive physicochemical characterization.

### 7.9 Pharmacokinetic and drug-likeness profile

**FIGURE 11:**
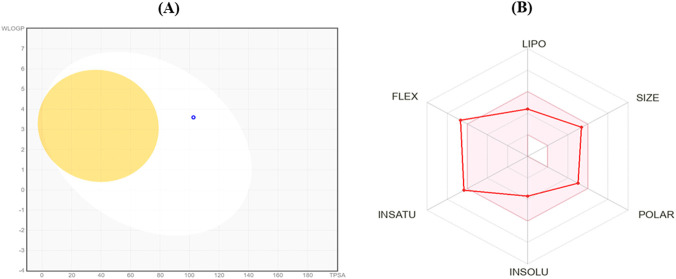
In silico pharmacokinetic and drug-likeness profile of the WADDAICA-designed berberine-inspired analogue **(A)** BOILED-Egg plot representing predicted gastrointestinal (GI) absorption and blood–brain barrier (BBB) permeability. The compound is positioned within the white region, indicating high probability of passive GI absorption, while remaining outside the yolk region, suggesting limited BBB penetration. **(B)** Bioavailability radar summarizing physicochemical properties associated with oral drug-likeness, including lipophilicity (LIPO), molecular size (SIZE), polarity (POLAR), solubility (INSOLU), flexibility (FLEX), and saturation (INSATU). The profile falls within the optimal range for most parameters, supporting favorable pharmacokinetic characteristics and drug-likeness.

In silico ADMET analysis demonstrated that the WADDAICA-designed analogue exhibits high gastrointestinal absorption and limited blood–brain barrier permeability (Figure 11A), suggesting suitability for liver-targeted therapy. The bioavailability radar confirmed that key physicochemical parameters fall within optimal ranges for oral drug candidates (Figure 11B), supporting favorable pharmacokinetic properties.

### 7.10 *In Vitro* validation of cytoprotective and anti-steatotic effects

The biological efficacy of the WADDAICA-designed analogue was evaluated in a free fatty acid (FFA)-induced MASH-like HepG2 model. FFA treatment significantly reduced cell viability and increased lipid accumulation, confirming induction of steatotic stress.

**FIGURE 12:**
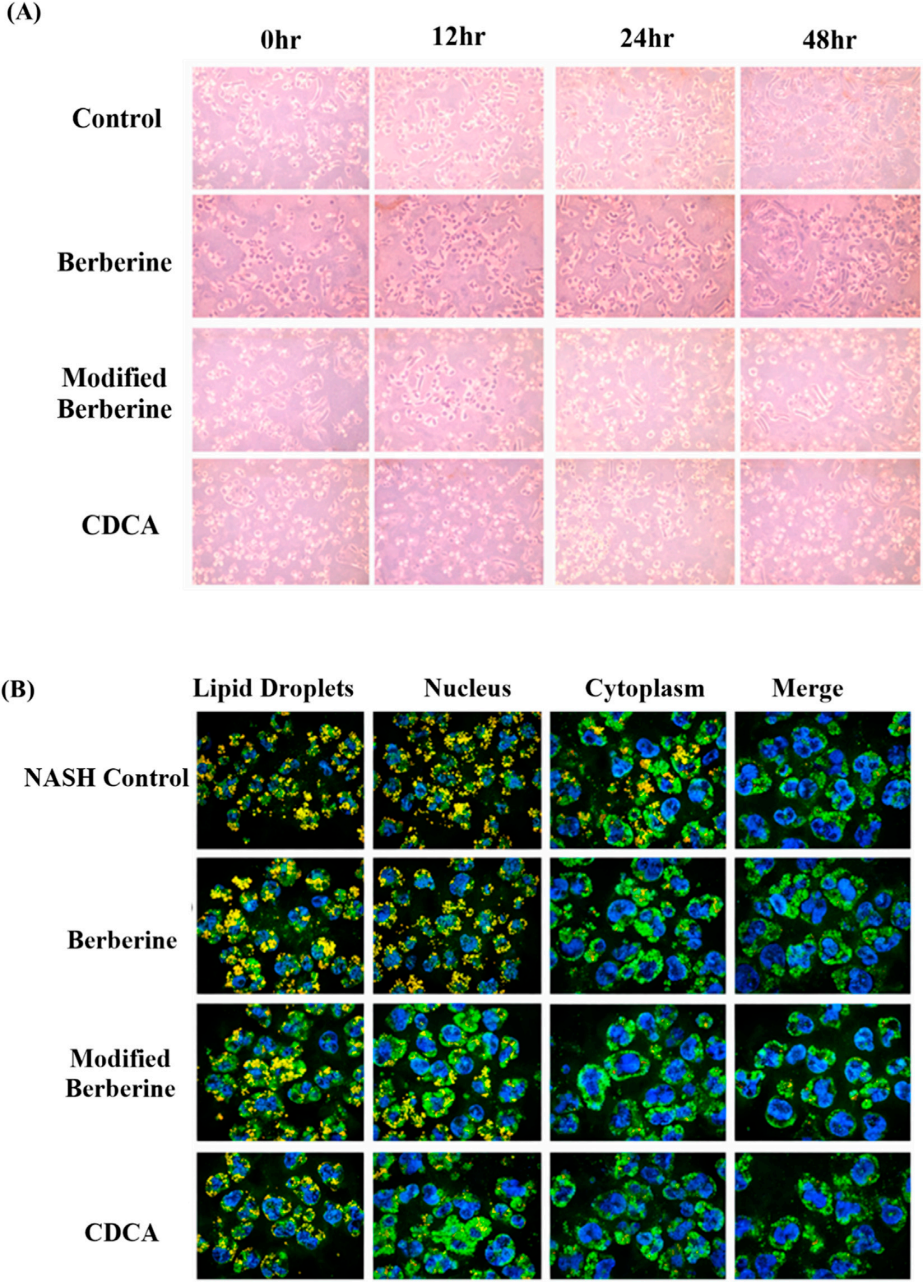
Cytoprotective and anti-steatotic effects of the WADDAICA-designed berberine-inspired analogue in a MASH-like HepG2 model **(A)** HepG2 cells were exposed to FFA (0.5 mM) for induction of steatosis-like cellular stress and subsequently treated with berberine, WADDAICA-designed berberine-inspired analogue, or CDCA at the indicated concentrations for 48 h. FFA-induced conditions result in reduced cell viability and altered cell morphology, whereas treatment with the WADDAICA-designed berberine-inspired analogue preserves cellular integrity and improves cell viability compared to native berberine. **(B)** Representative fluorescence images and quantitative analysis of intracellular lipid accumulation following FFA exposure (0.5 mM, 24–48 h) and subsequent compound treatment. Lipid accumulation was quantified from fluorescence intensity measurements using ImageJ.

Treatment with the WADDAICA-designed berberine-inspired analogue significantly restored cell viability (85.2% ± 2.4%) compared to FFA-treated cells (54.6% ± 2.9%), demonstrating strong cytoprotective effects (Table 7; Supplementary Table S4; Figure 12A). Additionally, fluorescence microscopy revealed a marked reduction in intracellular lipid accumulation following treatment, indicating attenuation of the steatotic phenotype (Figure 12B).

**TABLE 7:** Functional validation of the WADDAICA-designed berberine-inspired analogue in a MASH-like HepG2 model: effects on cell viability, lipid accumulation, and nuclear integrity.

Treatment	Cell viability (%) (48 h)	Lipid intensity (AU)	Nuclear integrity (%)
Control	100.0 ± 2.6	1.00 ± 0.06	98.5 ± 1.2
FFA (MASH)	54.6 ± 2.9***	2.91 ± 0.14***	71.3 ± 2.8***
Berberine	68.5 ± 3.0**	2.05 ± 0.11**	82.6 ± 2.5**
Berberine-inspired analogue	85.2 ± 2.4###	1.29 ± 0.09###	93.2 ± 1.9###
CDCA	90.8 ± 2.2###	1.16 ± 0.07###	95.6 ± 1.7###

Data are presented as mean ± SD, from three independent biological replicates (n = 3). Statistical comparisons were performed using one-way ANOVA, followed by Tukey’s multiple-comparison test. *p < 0.05, **p < 0.01, ***p < 0.001 versus Control; #p < 0.05, ##p < 0.01, ###p < 0.001 versus FFA-treated cells.

### 7.11 Modulation of FXR-associated molecular pathways

**FIGURE 13:**
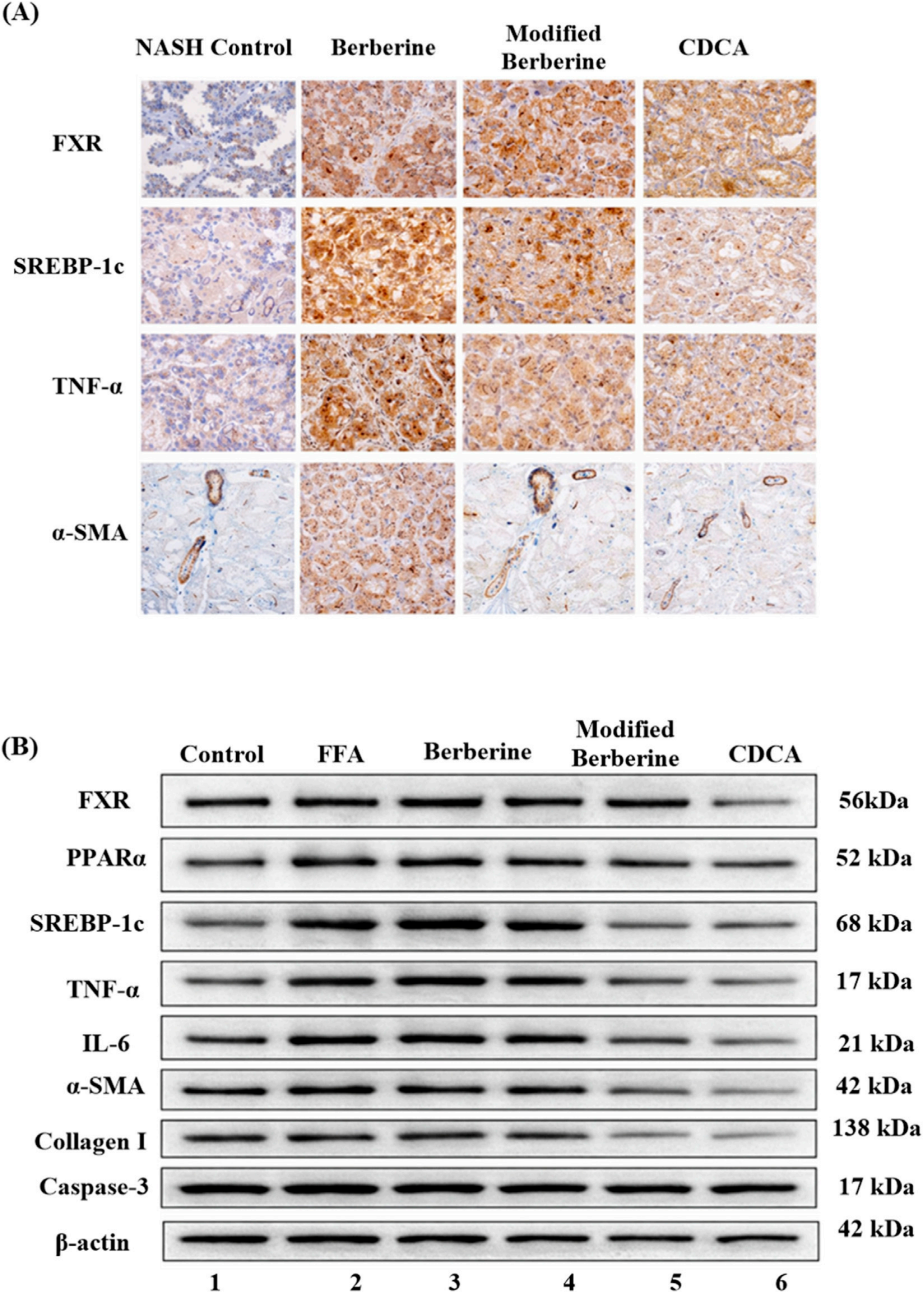
Modulation of FXR-associated metabolic, inflammatory, and fibrotic markers by the WADDAICA-designed berberine-inspired analogue in a MASH-like model **(A)** Immunohistochemical (IHC) analysis of key markers in HepG2 cells under MASH-like conditions. FFA treatment reduces FXR expression and increases lipogenic (SREBP-1c), inflammatory (TNF-α), and fibrotic (α-SMA) markers. Treatment with the WADDAICA-designed berberine-inspired analogue restores FXR expression while suppressing SREBP-1c, TNF-α, and α-SMA levels, indicating reversal of metabolic dysregulation, inflammation, and fibrosis. **(B)** Western blot analysis of protein expression levels for FXR, PPARα, SREBP-1c, TNF-α, IL-6, α-SMA, collagen I, and caspase-3 across treatment groups. The WADDAICA-designed berberine-inspired analogue upregulates FXR and PPARα expression while downregulating lipogenic, inflammatory, fibrotic, and apoptotic markers compared to FFA-treated cells. β-actin was used as a loading control.

Western blot analysis further confirmed upregulation of FXR and PPARα, along with downregulation of inflammatory markers (TNF-α and IL-6), fibrosis-associated markers (α-SMA and collagen I), and apoptotic marker cleaved caspase-3 (Figure 13; Supplementary Figure S5A).

To clarify the variability reported in Table 8, all quantitative expression values were calculated from independent biological replicates after normalization to the corresponding internal controls. Western blot band intensities were normalized to β-actin, qPCR data were normalized to GAPDH using the 2^−ΔΔCT^ method, and immunocytochemistry fluorescence intensities were quantified under identical imaging settings and normalized to the control group. Therefore, the reported SD values represent variation among normalized biological-replicate fold-change values rather than variation in raw band intensities, raw Ct values, raw fluorescence values, or pooled technical measurements. The relatively narrow SD range reflects normalized fold-change data obtained under controlled *in vitro* conditions and should not be interpreted as variability in raw experimental signals.

**TABLE 8:** Normalized protein and gene-expression changes in FFA-induced HepG2 cells after treatment with berberine, the WADDAICA-designed berberine-inspired analogue, and CDCA.

Marker/Gene	Level	Control	FFA	Berberine	Berberine-inspired analogue	CDCA
FXR (NR1H4)	ICC	1.00 ± 0.05	0.50 ± 0.04***	0.86 ± 0.06**	1.48 ± 0.08###	1.60 ± 0.07###
	WB	1.00 ± 0.05	0.51 ± 0.04***	0.87 ± 0.06**	1.44 ± 0.07###	1.58 ± 0.06###
	qPCR	1.00 ± 0.06	0.47 ± 0.05***	0.83 ± 0.07**	1.47 ± 0.09###	1.62 ± 0.08###
PPARα (PPARA)	WB	1.00 ± 0.06	0.60 ± 0.05***	0.93 ± 0.07**	1.36 ± 0.08###	1.48 ± 0.07###
	qPCR	1.00 ± 0.05	0.54 ± 0.04***	0.91 ± 0.06**	1.34 ± 0.08###	1.51 ± 0.07###
SREBP-1c (SREBP1)	ICC	1.00 ± 0.06	2.10 ± 0.11***	1.48 ± 0.09**	1.12 ± 0.07###	0.98 ± 0.06###
	WB	1.00 ± 0.07	1.98 ± 0.12***	1.45 ± 0.09**	1.10 ± 0.08###	0.96 ± 0.06###
	qPCR	1.00 ± 0.06	2.12 ± 0.13***	1.52 ± 0.10**	1.08 ± 0.08###	0.97 ± 0.06###
TNF-α (TNFA)	ICC	1.00 ± 0.05	2.35 ± 0.12***	1.78 ± 0.10**	1.22 ± 0.08###	1.05 ± 0.07###
	WB	1.00 ± 0.05	2.32 ± 0.14***	1.76 ± 0.10**	1.21 ± 0.08###	1.06 ± 0.07###
	qPCR	1.00 ± 0.05	2.41 ± 0.15***	1.82 ± 0.11**	1.27 ± 0.09###	1.06 ± 0.07###
IL-6 (IL6)	WB	1.00 ± 0.06	2.12 ± 0.13***	1.63 ± 0.11**	1.16 ± 0.09###	1.04 ± 0.08###
	qPCR	1.00 ± 0.06	2.28 ± 0.14***	1.71 ± 0.12**	1.22 ± 0.10###	1.09 ± 0.08###
α-SMA (ACTA2)	ICC	1.00 ± 0.06	2.20 ± 0.13***	1.65 ± 0.11**	1.18 ± 0.09###	1.06 ± 0.08###
	WB	1.00 ± 0.07	2.26 ± 0.15***	1.68 ± 0.12**	1.20 ± 0.09###	1.08 ± 0.07###
	qPCR	1.00 ± 0.06	2.22 ± 0.14***	1.67 ± 0.12**	1.24 ± 0.09###	1.11 ± 0.08###
Collagen I (COL1A1)	WB	1.00 ± 0.06	2.45 ± 0.16***	1.90 ± 0.13**	1.28 ± 0.10###	1.12 ± 0.08###
	qPCR	1.00 ± 0.07	2.52 ± 0.16***	1.92 ± 0.13**	1.32 ± 0.11###	1.14 ± 0.09###
Caspase-3	WB	1.00 ± 0.05	2.08 ± 0.14***	1.57 ± 0.11**	1.12 ± 0.09###	1.02 ± 0.07###
CYP7A1	qPCR	1.00 ± 0.05	0.59 ± 0.04***	0.94 ± 0.07**	1.42 ± 0.08###	1.57 ± 0.07###

Data are presented as mean ± SD, from three independent biological replicates. For ICC, fluorescence intensity was quantified under identical imaging settings and normalized to the control group. For Western blotting, target-protein band intensity was normalized to β-actin before fold-change calculation. For qPCR, gene expression was normalized to GAPDH, using the 2^−ΔΔCT^, method. SD, values were calculated from normalized biological-replicate values and not from pooled technical replicates or raw signal values. The relatively narrow SD, range reflects variation in normalized fold-change values under controlled *in vitro* conditions. Statistical comparisons were performed using one-way ANOVA, followed by Tukey’s *post hoc* test. *p < 0.05, **p < 0.01, ***p < 0.001 versus Control; #p < 0.05, ##p < 0.01, ###p < 0.001 versus FFA-treated cells.

**FIGURE 14:**
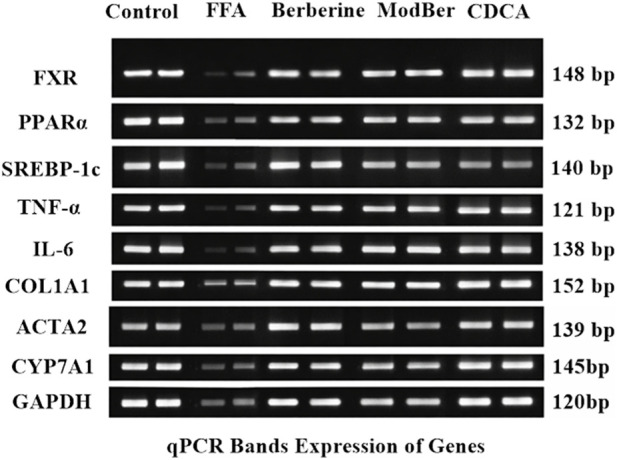
Gene expression analysis of FXR-regulated metabolic, inflammatory, and fibrotic markers by qPCR. Reverse transcription PCR (RT-PCR) analysis of gene expression in HepG2 cells under control, FFA-induced MASH-like conditions, and compound-treated groups. FFA treatment reduces FXR (NR1H4), PPARα, and CYP7A1 expression while upregulating lipogenic (SREBP1), inflammatory (TNFA, IL6), and fibrotic (COL1A1, ACTA2) genes. Treatment with the WADDAICA-designed berberine-inspired analogue restores FXR pathway gene expression and suppresses downstream pathological markers compared to FFA-treated cells. GAPDH was used as an internal control for normalization.

Quantitative densitometry and gene-expression results are summarized in Table 8. Gene-expression analysis using qPCR showed consistent trends, with restoration of FXR (NR1H4), PPARA, and CYP7A1 expression and suppression of SREBP1, TNFA, IL6, COL1A1, and ACTA2 (Figure 14; Supplementary Figure S5B; Table 8).

### 7.12 Integrated multi-scale analysis and mechanistic interpretation

An integrated summary of computational and experimental findings is presented in Tables 9, 10. Computational analyses supported favorable predicted interaction with FXR binding, structural stability, and favorable pharmacokinetic properties, while experimental validation confirmed cytoprotective, anti-steatotic, and anti-inflammatory effects and demonstrated modulation of fibrosis-associated molecular markers in the FFA-induced HepG2 model.

**TABLE 9:** Integrated summary table. Computational analyses supporting FXR-associated biological activity of the WADDAICA-designed berberine-inspired analogue.

Category	Parameter	Result	Interpretation	Relevance
Chemical characterization	HPLC	Dominant chromatographic peak; purity 98.6%	Chromatographic purity	Supports sample purity, not molecular identity
Chemical characterization	FTIR	N–H, amide-associated C=O, aromatic C=C, C–O–C, and C–F bands	Supportive functional-group evidence	Not used as sole structural proof
Chemical characterization	TLC	Product spot distinct from starting material	Reaction monitoring	Not used for molecular identity confirmation
Chemical characterization	NMR/LC–MS	Consistent spectral and molecular-ion data	Primary structural evidence	Supports proposed molecular identity

• Computational and analytical parameters are reported as representative outputs from validated models and experimental characterization methods.

**TABLE 10:** Integrated summary table. Quantitative summary of biological effects of the WADDAICA-designed berberine-inspired analogue in a MASH-like model.

Category	Marker	Control	FFA	Berberine-inspired analogue	Statistical significance	Interpretation
Cell viability	MTT (%)	100.0 ± 2.3	54.6 ± 2.9	85.2 ± 2.4	###	Cytoprotective effect
Lipid accumulation	Lipid intensity	1.00 ± 0.06	2.91 ± 0.14	1.29 ± 0.09	###	Anti-steatotic
Protein expression	FXR	1.00 ± 0.05	0.51 ± 0.04	1.44 ± 0.07	###	Pathway activation
	PPARα	1.00 ± 0.06	0.60 ± 0.05	1.36 ± 0.08	###	Lipid metabolism ↑
	SREBP-1c	1.00 ± 0.07	1.98 ± 0.12	1.10 ± 0.08	###	Lipogenesis ↓
	TNF-α	1.00 ± 0.05	2.32 ± 0.14	1.21 ± 0.08	###	Inflammation ↓
	IL-6	1.00 ± 0.06	2.12 ± 0.13	1.16 ± 0.09	###	Inflammation ↓
	α-SMA	1.00 ± 0.07	2.26 ± 0.15	1.20 ± 0.09	###	Fibrosis ↓
	Collagen I	1.00 ± 0.06	2.45 ± 0.16	1.28 ± 0.10	###	Fibrosis ↓
Gene expression	FXR (NR1H4)	1.00 ± 0.06	0.47 ± 0.05	1.47 ± 0.09	###	Pathway activation
	PPARA	1.00 ± 0.05	0.54 ± 0.04	1.34 ± 0.08	###	Lipid metabolism ↑
	SREBP1	1.00 ± 0.06	2.12 ± 0.13	1.08 ± 0.08	###	Lipogenesis ↓
	TNFA	1.00 ± 0.05	2.41 ± 0.15	1.27 ± 0.09	###	Anti-inflammatory
	IL6	1.00 ± 0.06	2.28 ± 0.14	1.22 ± 0.10	###	Anti-inflammatory
	COL1A1	1.00 ± 0.07	2.52 ± 0.16	1.32 ± 0.11	###	Anti-fibrotic
	ACTA2	1.00 ± 0.06	2.22 ± 0.14	1.24 ± 0.09	###	Anti-fibrotic
	CYP7A1	1.00 ± 0.05	0.59 ± 0.04	1.42 ± 0.08	###	Bile acid metabolism ↑

• Values are presented as mean ± standard deviation (SD) from three independent experiments.

• Statistical significance was determined using one-way ANOVA followed by Tukey’s post hoc test.

• * p < 0.05 vs Control; ** p < 0.01 vs Control; *** p < 0.001 vs Control

• #p < 0.05 vs FFA; ## p < 0.01 vs FFA; ### p < 0.001 vs FFA

• FXR: Farnesoid X receptor; PPARα: Peroxisome proliferator-activated receptor alpha;

SREBP-1c: Sterol regulatory element-binding protein 1c; TNF-α: Tumor necrosis factor alpha; IL-6: Interleukin-6; α-SMA: Alpha-smooth muscle actin; COL1A1: Collagen type I alpha 1; CYP7A1: Cholesterol 7 alpha-hydroxylase.

Collectively, these findings demonstrate that WADDAICA-guided design of a berberine-inspired analogue yields a compound with enhanced FXR-associated biological activity and improved biological efficacy.

### 7.13 Proposed mechanism of action

**FIGURE 15:**
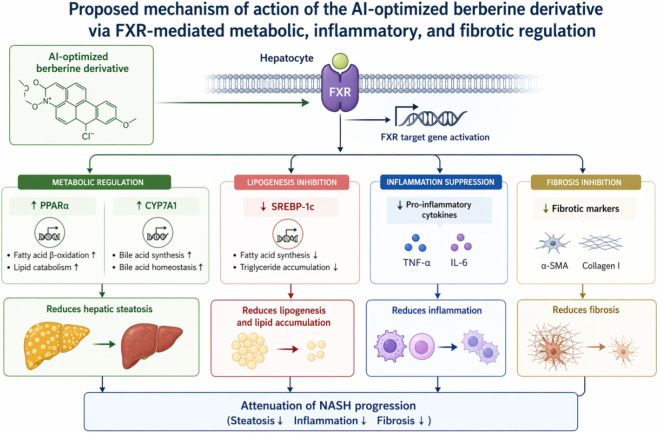
Proposed FXR-mediated mechanism of the WADDAICA-designed berberine-inspired analogue in a MASH model. Schematic representation of the proposed mechanism underlying the therapeutic effects of the WADDAICA-designed berberine-inspired analogue in a MASH-like system. The compound activates the farnesoid X receptor (FXR), a key nuclear regulator of hepatic metabolism, leading to transcriptional upregulation of PPARα and CYP7A1, which promote lipid catabolism and bile acid homeostasis. Concurrently, FXR-associated pathway modulation suppresses lipogenic signaling via SREBP-1c, reduces pro-inflammatory cytokines (TNF-α, IL-6), and inhibits fibrotic markers (α-SMA, collagen I). These coordinated regulatory effects result in attenuation of hepatic steatosis, inflammation, and fibrosis, ultimately reducing MASH progression.

The mechanistic basis of the observed therapeutic effects is illustrated in Figure 15. The observed biological effects are consistent with modulation of FXR-associated signaling pathways. The WADDAICA-designed analogue was associated with increased FXR expression and regulation of downstream metabolic markers, including PPARα, CYP7A1, and SREBP-1c. While these findings support a potential FXR-mediated mechanism, direct receptor activation was not experimentally demonstrated in the present study and therefore requires confirmation using dedicated functional assays.

Simultaneously, FXR-associated pathway modulation suppresses SREBP-1c-mediated lipogenesis and reduces inflammatory cytokines (TNF-α, IL-6) and fibrotic markers (α-SMA, collagen I). These coordinated effects result in attenuation of hepatic steatosis, inflammation, and fibrosis, thereby reducing MASH progression.

## 8 Discussion

A key clarification of the present study is that the workflow does not represent an end-to-end AI model that converts clinical variables into optimized chemical structures. The SE attention-based Transformer model and the WADDAICA molecular-design platform served distinct functions. The Transformer model was trained on clinical biochemical data for liver-disease classification and feature-importance analysis. In contrast, WADDAICA was used independently for molecular design based on chemical and receptor-related criteria. Therefore, the clinical diagnostic dataset was not used to train a molecular generator or compound optimizer. This distinction is essential because diagnostic clinical datasets and compound-optimization datasets have fundamentally different input-output structures (Mi et al., 2003).

### 8.1 AI-driven feature extraction and biological relevance

The SE attention-based Transformer model demonstrated high predictive performance, reflecting its ability to capture nonlinear relationships among clinical variables. Importantly, the identification of bilirubin fractions, albumin, and transaminases as key predictive features provides biological credibility to the model. These biomarkers are closely linked to hepatic metabolic regulation and are known to be influenced by FXR signaling pathways (Esteva et al., 2019; Chiang, 2013).

A major limitation of many AI-based clinical studies is the lack of interpretability and biological grounding (Esteva et al., 2019; Jiao et al., 2015; Samek and Müller, 2019). The use of SE attention mechanisms in this study enabled dynamic prioritization of clinically relevant features, enhancing interpretability while maintaining predictive accuracy. This is consistent with recent reports highlighting the utility of attention-based architectures in biomedical applications (Esteva et al., 2019; Samek and Müller, 2019; Hu et al., 2020). However, unlike previous studies that remain confined to prediction, the present work extends AI-derived insights toward actionable biological target identification, representing a critical advancement.

### 8.2 FXR as a mechanistic link between metabolism and disease

The selection of FXR as a central regulatory node is supported by its well-established role in hepatic metabolism, bile acid homeostasis, and inflammatory signaling. FXR functions as a key integrator of metabolic pathways, regulating lipid synthesis, fatty acid oxidation, and bile acid transport (Hu et al., 2020; Panzitt et al., 2025). The observed protein–protein interaction networks and gene co-expression patterns further reinforce its central role in coordinating hepatic metabolic responses.

Previous studies have demonstrated that FXR-associated pathway modulation reduces hepatic steatosis, suppresses inflammation, and attenuates fibrosis, making it a promising therapeutic target for MASH (Watanabe et al., 2006; Kydd-Sinclair et al., 2025). However, clinical FXR modulators such as obeticholic acid are associated with adverse effects and inconsistent efficacy (Kremoser, 2021). These challenges highlight the need for alternative modulators with improved safety and specificity, which the current study aims to address through rational compound optimization.

### 8.3 Rational design and optimization of berberine derivative

Berberine has been widely studied for its metabolic benefits; however, its clinical application is limited by poor bioavailability and lack of target specificity (Neuschwander-Tetri et al., 2015; Filli et al., 2022). In this study, WADDAICA-guided molecular design resulted in a berberine-inspired analogue derivative with improved physicochemical properties and pharmacokinetic profile. The enhanced lipophilicity and favorable ADMET characteristics are consistent with previous reports indicating that structural modification can improve the pharmacological performance of natural compounds (Shi et al., 2025; Naz et al., 2022).

Molecular docking predicted favorable ligand–receptor interactions between the WADDAICA-designed analogue and the FXR ligand-binding domain. While docking scores provide useful indications of potential binding behavior, they should be interpreted as computational predictions rather than quantitative measures of experimental binding affinity (Watanabe et al., 2006; Mushtaq et al., 2023).

Molecular dynamics (MD) simulations further confirmed the stability of the ligand–receptor complex, with consistent RMSD, radius of gyration, and hydrogen bond profiles. Such stability is indicative of sustained receptor engagement, which is critical for pharmacological activity (Yang et al., 2024; Hollingsworth and Dror, 2018). The integration of MD simulations with docking provides a more reliable assessment of ligand behavior compared to static models alone (Yang et al., 2024).

Importantly, DFT calculations evaluate electronic structure and molecular reactivity rather than biological activity and should therefore be regarded as supplementary physicochemical analysis (Karplus and McCammon, 2002; Blanchard and Brüning, 2015).

### 8.4 Biological validation and functional relevance

The *in vitro* findings provide strong validation of the computational predictions. In the FFA-induced MASH-like model, the WADDAICA-designed analogue significantly improved cell viability and reduced lipid accumulation, indicating attenuation of steatotic stress. These observations are consistent with previous studies demonstrating the anti-steatotic and hepatoprotective effects of FXR-associated pathway modulation and berberine derivatives (Kydd-Sinclair et al., 2025; Geerlings et al., 2014). Nevertheless, the FFA-induced HepG2 system represents a simplified *in vitro* model that primarily captures steatosis-associated metabolic stress and does not fully reflect the multicellular pathophysiology of MASH.

At the molecular level, the upregulation of FXR and PPARα, along with downregulation of SREBP-1c, TNF-α, IL-6, α-SMA, and collagen I, confirms coordinated regulation of metabolic, inflammatory, and fibrotic pathways. These results are in agreement with established mechanisms of FXR-mediated metabolic control and further validate the therapeutic relevance of the WADDAICA-designed analogue (Hu et al., 2020; Wang et al., 2024). Although the observed molecular changes are consistent with FXR pathway involvement, the present study did not directly evaluate putative FXR agonism or receptor activation. Therefore, the findings should be interpreted as evidence of FXR-associated pathway modulation rather than definitive proof of direct FXR agonism or receptor-specific activation.

Importantly, the consistency between protein expression (Western blot and immunocytochemistry) and gene expression (qPCR) enhances the reliability of the findings and reduces the likelihood of experimental bias. Multi-level validation is increasingly recognized as essential for translational research, particularly in complex diseases such as MASH (Cariou and Staels, 2007).

### 8.5 Mechanistic integration and translational implications

The integrated analysis supports a mechanistic model in which the WADDAICA-designed analogue may influence FXR-associated pathways, leading to improved lipid metabolism and reduced inflammatory signaling. This multi-target regulatory effect is particularly relevant for MASH, which involves interconnected pathological processes rather than a single molecular defect (Venhorst et al., 2024).

The novelty of this study lies in the seamless integration of AI-driven clinical insights with molecular and biological validation. Unlike conventional approaches that examine drug candidates or targets independently, this work establishes a direct link between clinical data, target identification, and therapeutic design. Such integrative frameworks are increasingly recognized as essential for accelerating drug discovery and improving translational success rates (Tilg et al., 2017).

### 8.6 Limitations

Despite the strengths of this study, several limitations should be acknowledged. First, the AI model was developed using a structured clinical dataset and requires validation in independent, large-scale, and multi-center cohorts to establish its generalizability and clinical applicability (Bai et al., 2021; Lavecchia, 2019). Second, although molecular docking, molecular dynamics simulations, MM-GBSA calculations, and DFT analyses provided valuable computational insights, these approaches are inherently predictive and may not fully capture the complexity of biological systems or accurately reflect experimental binding behavior (Yang et al., 2024).

Third, experimental validation was performed in an FFA-induced HepG2 MASH-like model, which represents a simplified *in vitro* system and does not fully reproduce the multicellular, inflammatory, immunological, and fibrotic complexity of human MASH (Beam and Kohane, 2018). In addition, assessment of α-SMA and collagen I was conducted in HepG2 cells rather than hepatic stellate cells, the primary mediators of liver fibrosis. Therefore, changes in these markers should be interpreted as preliminary evidence of modulation of fibrosis-associated signaling rather than definitive proof of anti-fibrotic activity. Future studies should incorporate hepatic stellate cell models, hepatocyte–stellate cell co-culture systems, liver organoids, and *in vivo* MASH models to more rigorously evaluate therapeutic efficacy.

Furthermore, direct functional assessment of FXR activity was not performed. While computational analyses together with gene and protein expression studies support the involvement of FXR-associated pathways, they do not establish direct receptor binding or agonism. Future investigations should include FXR reporter assays, antagonist competition studies, coactivator recruitment assays, and genetic knockdown approaches to verify receptor-specific activity and mechanism of action.

Another limitation is that the clinical AI model and molecular-design platform were not integrated into a single end-to-end drug-discovery model. The Indian Liver Patient Dataset is suitable for diagnostic classification and feature interpretation, but it lacks molecular structures, compound-target activity labels, receptor-binding affinities, pharmacokinetic endpoints, and chemical descriptors. Therefore, it cannot directly support compound optimization. Future studies should integrate disease-specific clinical datasets with compound bioactivity, transcriptomic, metabolomic, and ligand-target interaction datasets to develop a fully integrated AI-guided therapeutic design framework.

A limitation of the original presentation was that the origin and structural verification of the WADDAICA-designed analogue were not described with sufficient chemical detail. Because the *in vitro* expression analyses were based on three biological replicates, the quantitative results should be interpreted as preliminary validation. Future studies should confirm these molecular trends using larger replicate numbers and independent experimental repeats.

Finally, MASH is a multifactorial disease involving numerous interconnected metabolic, inflammatory, and fibrotic pathways. Therefore, modulation of FXR-associated signaling alone may not fully account for the observed biological effects or completely address all aspects of disease progression (Venhorst et al., 2024; Anstee et al., 2019).

### 8.7 Future directions

Future studies should focus on validating the AI model using diverse clinical datasets to improve robustness and clinical applicability. *In vivo* studies using relevant animal models are necessary to evaluate the pharmacokinetics, efficacy, and safety of the WADDAICA-designed analogue. Further medicinal chemistry optimization may enhance target specificity and bioavailability.

tIntegration of multi-omics approaches, including transcriptomics and metabolomics, could provide deeper insights into disease mechanisms and therapeutic responses. Additionally, the incorporation of explainable AI techniques may improve model transparency and facilitate clinical translation (Chiang, 2013; Tilg et al., 2017).

## 9 Conclusion

This study presents a sequential clinical-to-molecular framework in which an SE attention-based Transformer model was used for liver-disease classification and clinical feature learning, while WADDAICA was used independently for the design of a berberine-inspired analogue predicted to interact with FXR-associated pathways in MASH. The WADDAICA-designed berberine-inspired analogue demonstrated favorable computational interaction profiles with FXR, promising pharmacokinetic characteristics, and beneficial biological effects in a preliminary FFA-induced HepG2 model, including improved cell viability, reduced lipid accumulation, and modulation of metabolic and inflammatory markers. By integrating clinical prediction, computational modeling, and experimental validation, this work establishes a multi-scale platform for precision drug discovery in metabolic liver disease. While the computational and biological findings support the potential involvement of FXR-related signaling, direct FXR activation was not experimentally confirmed and requires further investigation using dedicated receptor functional assays. Moreover, validation in advanced cellular systems and *in vivo* MASH models will be necessary to establish therapeutic efficacy and mechanism of action. Overall, the study highlights the potential of AI-guided approaches to accelerate the discovery and optimization of candidate therapeutics for complex metabolic disorders.

## Data Availability

The datasets presented in this study can be found in online repositories. The names of the repository/repositories and accession number(s) can be found in the article/Supplementary Material.
