# Supplementary material for: Clinical AI-assisted FXR prioritization and WADDAICA-guided design of a berberine-inspired analogue for MASH: integrated computational and experimental validation
Source: Front Pharmacol. 2026 Jul 23;17:1862990. doi: 10.3389/fphar.2026.1862990 (PMC13443328; doi:10.3389/fphar.2026.1862990)
Supplement: Supplementary file 1 [file Supplementaryfile1.docx]

## **SUPPLEMENTARY MATERIALS**

## **Table S1. Natural compound library screened for FXR-targeted drug discovery**

| **Compound** | **Natural Source** | **PubChem CID** |
| --- | --- | --- |
| Quercetin | Onions, apples | 5280343 |
| Kaempferol | Tea, broccoli | 5280863 |
| Luteolin | Celery, parsley | 5280445 |
| Apigenin | Chamomile, parsley | 5280443 |
| Naringenin | Citrus fruits | 932 |
| Hesperetin | Citrus fruits | 72281 |
| Curcumin | Turmeric | 969516 |
| Resveratrol | Grapes, berries | 445154 |
| Ursolic acid | Apple peel, herbs | 64945 |
| Glycyrrhetinic acid | Licorice root | 10114 |
| Betulinic acid | Birch bark | 64971 |
| Berberine | Berberis species | 2353 |
| Chlorogenic acid | Coffee beans | 1794427 |
| Ferulic acid | Grains, rice bran | 445858 |
| Catechin | Tea, cocoa, grapes | 9064 |
| Epigallocatechin gallate (EGCG) | Green tea | 65064 |
| Rutin | Citrus peels, buckwheat | 5280805 |
| Fisetin | Strawberries, apples, onions | 5281614 |
| Genistein | Soybeans | 5280961 |
| Daidzein | Soybeans, legumes | 5281708 |
| Ellagic acid | Pomegranate, berries | 5281855 |
| Gallic acid | Tea leaves | 370 |
| Rosmarinic acid | Rosemary, sage | 5281792 |
| Thymoquinone | Nigella sativa | 10281 |
| Piperine | Black pepper | 638024 |
| Chenodeoxycholic acid (CDCA) | Endogenous ligand | 10133 |
| Obeticholic acid (OCA) | Clinical Putative FXR modulator | 447715 |

- Natural compounds with reported hepatoprotective, anti-inflammatory, or metabolic regulatory properties were selected for virtual screening against the farnesoid X receptor (FXR). Chenodeoxycholic acid (CDCA) and obeticholic acid (OCA) were included as endogenous and clinically validated FXR modulators, respectively. Berberine was selected as the lead compound for further optimization based on its favorable docking and pharmacokinetic profile.
- **PubChem CID**: Unique compound identifier retrieved from the PubChem database.
- **EGCG**: Epigallocatechin gallate.
- **CDCA**: Chenodeoxycholic acid.
- **OCA**: Obeticholic acid (INT-747).
- Natural sources are representative and not exhaustive.

# **Table S2. Comprehensive docking and pharmacokinetic profiling of natural compounds targeting FXR**

| **Compound** | **Binding Energy (kcal/mol)** | **Solubility** | **GI Absorption** | **BBB** | **Lipinski (violations)** | **Toxicity Class** |
| --- | --- | --- | --- | --- | --- | --- |
| Chenodeoxycholic acid (CDCA) | −10.8 | Soluble | High | No | ✔ (0) | Class 4 |
| Berberine | −9.2 | Moderately soluble | High | Yes | ✔ (0) | Class 3 |
| Betulinic acid | −9.2 | Poorly soluble | Low | No | ✔ (1) | Class 5 |
| Obeticholic acid (OCA) | −9.0 | Moderately soluble | High | No | ✔ (1) | Class 4 |
| Curcumin | −8.7 | Soluble | High | No | ✔ (0) | Class 4 |
| Genistein | −8.5 | Soluble | High | No | ✔ (0) | Class 5 |
| Piperine | −8.5 | Soluble | High | Yes | ✔ (0) | Class 4 |
| Rutin | −8.5 | Soluble | Low | No | ✘ (3) | Class 5 |
| Epigallocatechin gallate (EGCG) | −8.4 | Soluble | Low | No | ✘ (2) | Class 4 |
| Apigenin | −8.3 | Soluble | High | No | ✔ (0) | Class 5 |
| Fisetin | −8.3 | Soluble | High | No | ✔ (0) | Class 3 |
| Hesperetin | −8.3 | Soluble | High | No | ✔ (0) | Class 4 |
| Luteolin | −8.3 | Soluble | High | No | ✔ (0) | Class 5 |
| Quercetin | −8.3 | Soluble | High | No | ✔ (0) | Class 3 |
| Ellagic acid | −8.2 | Soluble | High | No | ✔ (0) | Class 4 |
| Naringenin | −8.2 | Soluble | High | No | ✔ (0) | Class 4 |
| Catechin | −8.1 | Soluble | High | No | ✔ (0) | Class 6 |
| Chlorogenic acid | −8.1 | Very soluble | Low | No | ✔ (1) | Class 5 |
| Kaempferol | −8.1 | Soluble | High | No | ✔ (0) | Class 5 |
| Ursolic acid | −8.1 | Poorly soluble | Low | No | ✔ (1) | Class 4 |
| Daidzein | −8.0 | Soluble | High | Yes | ✔ (0) | Class 5 |
| Rosmarinic acid | −7.7 | Soluble | Low | No | ✔ (0) | Class 5 |
| Glycyrrhetinic acid | −7.3 | Poorly soluble | High | No | ✔ (1) | Class 4 |
| Resveratrol | −7.2 | Soluble | High | Yes | ✔ (0) | Class 4 |
| Thymoquinone | −6.5 | Soluble | High | Yes | ✔ (0) | Class 5 |
| Ferulic acid | −6.4 | Soluble | High | Yes | ✔ (0) | Class 4 |
| Gallic acid | −5.6 | Very soluble | High | No | ✔ (0) | Class 4 |

- Comprehensive molecular docking and in silico pharmacokinetic profiling of natural compounds screened against the farnesoid X receptor (FXR). Binding affinities were calculated using AutoDock Vina, while solubility, absorption, and drug-likeness parameters were predicted using SwissADME. Toxicity classes were estimated using ProTox-II. Reference compounds include chenodeoxycholic acid (CDCA) and obeticholic acid (OCA), representing endogenous and clinically validated FXR modulators, respectively.
- **Binding Energy**: Predicted docking affinity (AutoDock Vina); more negative values indicate stronger binding.
- **Solubility**: Predicted aqueous solubility based on ESOL classification.
- **GI Absorption**: Predicted gastrointestinal absorption (High/Low).
- **BBB**: Predicted blood–brain barrier permeability.
- **Lipinski (violations)**: Number of violations of Lipinski’s Rule of Five; ✔ indicates compliance, ✘ indicates non-compliance.
- **Toxicity Class**: Predicted oral toxicity classification (ProTox-II; Class 1 = most toxic, Class 6 = least toxic).
- **EGCG**: Epigallocatechin gallate.
- All predictions were performed using SwissADME and ProTox-II platforms.

## **Table S3. Structural characterization and spectroscopic validation of the WADDAICA-designed berberine-inspired analogue**

### **(A) Synthesis and physicochemical characterization**

| **Parameter** | **Native Berberine** | **Berberine-inspired analogue** | **Interpretation** |
| --- | --- | --- | --- |
| Yield (%) | — | 72.4 | Efficient synthesis |
| Appearance | Yellow crystalline | Off-white solid | Structural modification |
| Purity (HPLC, %) | 97.1 | 98.6 | High purity achieved |
| Retention Time (min) | 4.82 | 6.37 | Increased hydrophobicity |
| Rf Value (TLC) | 0.42 | 0.61 | Chromatographic difference observed |

HPLC, TLC, and FTIR were used as supporting analytical methods. HPLC was used to assess chromatographic purity and retention behavior, TLC was used to monitor reaction progress, and FTIR was used to support functional-group assignment. These methods alone do not establish molecular identity. Structural confirmation of the WADDAICA-designed berberine-inspired analogue was based primarily on ¹H NMR, ¹³C NMR, and LC–MS analyses. The term “berberine-inspired analogue” is used because berberine served as the pharmacophoric reference during molecular design; the final compound should not be interpreted as a direct one-step chemical derivative of native berberine.

### **(B) FTIR spectral analysis**

| **Functional Group** | **Native Berberine (cm⁻¹)** | **Berberine-inspired analogue (cm⁻¹)** | **Assignment** |
| --- | --- | --- | --- |
| N–H stretch | — | 3325 | Amide formation |
| C=O (amide) | — | 1652 | Amide bond formation |
| Aromatic C=C | 1510 | 1522 | Aromatic framework |
| C–O–C stretch | 1265 | 1278 | Ether linkage |
| C–F stretch | — | 1105 | Fluorine substitution |

- Chemical characterization of the WADDAICA-designed berberine-inspired analogue demonstrating structural modification. High-performance liquid chromatography (HPLC) confirmed increased purity and altered retention time, indicating enhanced hydrophobicity. Thin-layer chromatography (TLC) further supported structural variation. Fourier-transform infrared (FTIR) spectroscopy revealed the introduction of amide functionality and fluorinated groups, confirming successful chemical modification of the berberine scaffold. These structural changes are consistent with suggested interaction potential toward the farnesoid X receptor (FXR).
- **HPLC**: High-performance liquid chromatography analysis performed using a C18 reverse-phase column.
- **TLC**: Thin-layer chromatography using silica gel plates.
- **Rf**: Retention factor calculated from TLC separation.
- **FTIR**: Fourier-transform infrared spectroscopy; values reported in wavenumbers (cm⁻¹).
- “—” indicates absence of the corresponding functional group in native berberine.
- All measurements were performed under standard laboratory conditions.

## **Table S4. Time-dependent effects of berberine and the WADDAICA-designed berberine-inspired analogue on HepG2 cell viability under MASH-like conditions**

| **Treatment** | **0 h (%)** | **12 h (%)** | **24 h (%)** | **48 h (%)** |
| --- | --- | --- | --- | --- |
| Control | 100.0 ± 1.8 | 100.0 ± 2.1 | 100.0 ± 2.3 | 100.0 ± 2.6 |
| FFA (MASH) | 98.6 ± 2.0 | 78.4 ± 2.8** | 61.8 ± 3.1*** | 54.6 ± 2.9*** |
| Berberine | 99.1 ± 1.7 | 88.2 ± 2.5* | 73.9 ± 2.7** | 68.5 ± 3.0** |
| Berberine-inspired analogue | 99.4 ± 1.5 | 94.6 ± 2.2### | 89.5 ± 2.1### | 85.2 ± 2.4### |
| CDCA | 99.6 ± 1.6 | 96.8 ± 2.0### | 94.1 ± 1.9### | 90.8 ± 2.2### |

- Data are presented as mean ± standard deviation (n = 3 independent experiments).
- **FFA (MASH)**: Free fatty acid-induced steatotic condition in HepG2 cells.
- **CDCA**: Chenodeoxycholic acid.
- Statistical analysis was performed using one-way analysis of variance (ANOVA) followed by Tukey’s post hoc test.
- ***** p < 0.05, ****** p < 0.01, ******* p < 0.001 vs Control group
- **#** p < 0.05, **##** p < 0.01, **###** p < 0.001 vs FFA (MASH) group

**Table S5: Assignment of ^1^H NMR signals for the synthesized berberine-inspired analogue derivative.**

| **1H NMR Assignment: Berberine** | | | | | | | | |
| --- | --- | --- | --- | --- | --- | --- | --- | --- |
| **Compound** | **Nucleus** | **Frequency (MHz)** | **δ / ppm** | **Integration** | **Multiplicity** | **J / Hz** | **Proposed assignment** | **Structural interpretation** |
| Berberine | 1H | 400 | 3.36 | 2H | ddd | 16.2, 6.8, 2.9 | Heterocyclic CH2 | Aliphatic methylene protons within reduced heterocyclic portion |
| Berberine | 1H | 400 | 3.60 | 3H | s |  | Ar–OCH3 | First aromatic methoxy substituent |
| Berberine | 1H | 400 | 3.81 | 3H | s |  | Ar–OCH3 | Second aromatic methoxy substituent |
| Berberine | 1H | 400 | 4.21 | 2H | ddd | 18.1, 6.8, 2.9 | N-adjacent heterocyclic CH2 | Deshielded methylene close to nitrogen-containing isoquinolinium system |
| Berberine | 1H | 400 | 6.45 | 2H | d | 12.6 | –OCH2O– | Methylenedioxy bridge attached to the aromatic ring |
| Berberine | 1H | 400 | 6.82 | 1H | dd | 8.9, 0.5 | Aromatic H | Proton of substituted aromatic ring |
| Berberine | 1H | 400 | 7.13–7.31 | 2H | overlapping signals | 7.18 d J=0.5; 7.26 d J=0.5 | Aromatic H | Fused aromatic ring protons of berberine skeleton |
| Berberine | 1H | 400 | 7.47 | 1H | ddd | 8.9, 2.0, 0.5 | Aromatic H | Deshielded aromatic proton in substituted fused ring system |
| Berberine | 1H | 400 | 7.71 | 1H | ddd | 2.0, 0.5, 0.5 | Aromatic H | Highly substituted aromatic proton |
| Berberine | 1H | 400 | 9.71 | 1H | dd | 0.5, 0.5 | Iminium/isoquinolinium proton | Diagnostic downfield proton of cationic berberine framework |

**Table S6: Assignment of ^13^C NMR signals for the synthesized berberine-inspired analogue derivative.**

| **13C NMR Assignment: Berberine** | | | | | | |
| --- | --- | --- | --- | --- | --- | --- |
| **Compound** | **Nucleus** | **δ / ppm** | **Carbon count** | **Multiplicity** | **Proposed assignment** | **Structural interpretation** |
| Berberine | 13C | 26.1 | 1C | s | Aliphatic CH2 | Heterocyclic methylene carbon |
| Berberine | 13C | 55 | 1C | s | Ar–OCH3 | Methoxy carbon |
| Berberine | 13C | 56.1 | 1C | s | Ar–OCH3 | Second methoxy carbon |
| Berberine | 13C | 61.4 | 1C | s | Heterocyclic/N-adjacent CH2 carbon | Deshielded methylene carbon near nitrogen-containing ring |
| Berberine | 13C | 101.6 | 1C | s | –OCH2O– / oxygenated aromatic region | Methylenedioxy-associated carbon environment |
| Berberine | 13C | 105.2 | 1C | s | Aromatic CH | Oxygen-substituted aromatic carbon environment |
| Berberine | 13C | 108.1 | 1C | s | Aromatic CH | Protonated aromatic carbon |
| Berberine | 13C | 120 | 1C | s | Aromatic CH/quaternary C | Fused aromatic ring carbon |
| Berberine | 13C | 120.2 | 1C | s | Aromatic CH/quaternary C | Fused aromatic ring carbon |
| Berberine | 13C | 121.2 | 1C | s | Aromatic CH | Aromatic carbon in isoquinoline framework |
| Berberine | 13C | 124.8 | 1C | s | Aromatic CH/quaternary C | Substituted aromatic carbon |
| Berberine | 13C | 125.1 | 1C | s | Aromatic CH/quaternary C | Substituted aromatic carbon |
| Berberine | 13C | 130.4 | 1C | s | Quaternary aromatic C | Fused ring junction carbon |
| Berberine | 13C | 132.9 | 1C | s | Quaternary aromatic C | Fused ring junction carbon |
| Berberine | 13C | 137.3 | 1C | s | Quaternary aromatic/iminium-associated C | Deshielded carbon of conjugated berberine system |
| Berberine | 13C | 145.2 | 1C | s | Oxygenated aromatic C | Ar–O carbon attached to methoxy/methylenedioxy substituent |
| Berberine | 13C | 146.7 | 1C | s | Oxygenated aromatic C | Ar–O carbon |
| Berberine | 13C | 146.9 | 1C | s | Oxygenated aromatic C | Ar–O carbon |
| Berberine | 13C | 147.5 | 1C | s | Oxygenated aromatic C | Ar–O carbon |
| Berberine | 13C | 149.6 | 1C | s | Highly deshielded aromatic C | Oxygenated/quaternary aromatic carbon |

**Table S7: Molecular ion and major fragment assignments obtained from ESI-LC-MS/MS analysis.**

| **ESI–LC–MS Molecular Ion Confirmation** | | | | | | | | | | |
| --- | --- | --- | --- | --- | --- | --- | --- | --- | --- | --- |
| **Compound** | **Assigned formula** | **Exact mass of cation (Da)** | **Average MW (g/mol)** | **Ionization mode** | **Assigned ion/adduct** | **Charge z** | **Observed m/z** | **Calculated m/z** | **Delta m/z** | **Key structural evidence** |
| Berberine | C20H18NO4(+1) | 336.1236 | 336.37 | Positive ESI | [M+H]2+ | 2 | 168.5652 | 168.5654 | -0.0002 | Parent protoberberine cation with methoxy and methylenedioxy fragments |
| Berberine-inspired analogue | C22H22F2N3O5(+1) | 446.1528 | 446.43 | Positive ESI | [M+H]2+ | 2 | 223.5797 | 223.58 | -0.0003 | Modified fluorinated pyrazolium–amide–carboxylated derivative |

**Table S8: HPLC retention time and chromatographic purity data for berberine and the berberine-inspired analogue derivative.**

| **LC Chromatographic Retention Time Results** | | | | | | | | |
| --- | --- | --- | --- | --- | --- | --- | --- | --- |
| **Compound** | **Chromatogram panel** | **Retention time tR (min)** | **Peak identity** | **Aligned molecular ion** | **Charge** | **Calculated exact mass (Da)** | **Mass spectral confirmation** | **Interpretation** |
| Berberine | First LC chromatogram / Panel A | 3.797 | Main LC peak | m/z 168.5652 | 2+ | 336.1236 | C20H18NO4(+1); [M+H]2+ in positive ESI | Sharp major peak aligned with parent berberine mass spectrum and methoxy/methylenedioxy fragmentation |
| Berberine-inspired analogue | Second LC chromatogram / Panel B | 3.956 | Main LC peak | m/z 223.5797 | 2+ | 446.1528 | C22H22F2N3O5(+1); [M+H]2+ in positive ESI | Slight later elution aligned with higher-mass modified derivative and diagnostic F-containing fragments |

**Figure S1. Training dynamics of the SE attention-based Transformer model over 100 epochs**

**(A)** Training and validation accuracy curves across 100 epochs, showing progressive improvement in model performance and convergence of training and validation accuracy.

**(B)** Training and validation loss curves over 100 epochs, demonstrating a gradual reduction in loss values and convergence between training and validation trends.

**Figure S2. Time evolution of molecular dynamics parameters and system energy for the FXR–WADDAICA-designed analogue complex**

**(A)** Time evolution of key molecular dynamics parameters over a 500 ns simulation, including root mean square deviation (RMSD), radius of gyration (Rg), intramolecular hydrogen bonds, molecular surface area (MolSA), solvent-accessible surface area (SASA), and polar surface area (PSA). The profiles indicate stable structural behavior and consistent physicochemical properties of the FXR–ligand complex throughout the simulation.

**(B)** Time evolution of total system energy (kcal/mol) for the FXR–WADDAICA-designed analogue complex over 500 ns, showing fluctuations within a stable range, indicative of thermodynamic stability under simulation conditions.

**Figure S3. Chromatographic and FTIR characterization of native berberine and the WADDAICA-designed berberine-inspired analogue.**

**(A) HPLC chromatograms of native berberine and the WADDAICA-designed berberine-inspired analogue showing different retention behavior and chromatographic purity. HPLC was used to assess purity and retention characteristics and was not used alone to establish molecular identity.**

**(B) FTIR spectra of native berberine and the berberine-inspired analogue showing characteristic functional-group absorptions. FTIR was used as supportive functional-group evidence and not as the sole method for structural confirmation.**

**Figure S4. TLC analysis of native berberine and the WADDAICA-designed berberine-inspired analogue.**

**TLC analysis was performed to monitor chromatographic behavior of native berberine and the WADDAICA-designed berberine-inspired analogue under visible and UV illumination. The difference in Rf values indicates chromatographic separation between the starting reference compound and the synthesized analogue. TLC was used only as a supporting reaction-monitoring method and not as definitive evidence of compound identity or purity.**

**Figure S5. Uncropped western blot and RT-PCR data supporting protein and gene expression analyses**

**(A)** Uncropped western blot images showing full-length protein bands for FXR, PPARα, SREBP-1c, TNF-α, IL-6, α-SMA, collagen I, and caspase-3 across experimental groups (Control, FFA, Berberine, Berberine-inspired analogue, CDCA). β-actin was used as a loading control. Molecular weights (kDa) are indicated for each protein.

**(B)** Uncropped RT-PCR gel images showing gene expression of FXR (NR1H4), PPARA, CYP7A1, SREBP1, TNFA, IL6, COL1A1, ACTA2, and GAPDH across experimental groups. DNA ladder (M) and product sizes (bp) are indicated.

These uncropped images are provided to ensure transparency and confirm that the processed data presented in the main figures were derived from full, unedited experimental outputs.

**Figure S6. Proposed synthetic route for the preparation of the WADDAICA-designed berberine-inspired analogue.**

Berberine was used as a pharmacophoric reference during molecular design. The proposed synthetic strategy involves activation of a carboxylic-acid intermediate using EDC-HCl and N-hydroxysuccinimide (NHS) in dimethylformamide (DMF), followed by coupling with an amine-containing fragment in the presence of triethylamine to generate the target analogue. The product was purified by silica-gel column chromatography and characterized using HPLC, FTIR, NMR, and LC–MS. TLC and HPLC were used to support reaction monitoring and chromatographic purity assessment, whereas NMR and LC–MS were used as the principal methods for structural confirmation.

**Figure S7:** ^1^H NMR spectrum of the synthesized berberine-inspired analogue derivative recorded in DMSO-d6 showing characteristic aromatic, methine, and linker proton resonances.

**Figure S8: ^1^H NMR spectrum of berberine used as reference for structural comparison with the synthesized derivative.**

**Figure S9: Positive-ion ESI-LC-MS/MS spectrum of the berberine-inspired analogue derivative showing the molecular ion peak and characteristic fragment ions supporting the proposed structure.**

**Figure S10: Representative HPLC chromatogram of the synthesized berberine-inspired analogue derivative demonstrating chromatographic purity.**
